# Multi source Earth observation and machine learning framework for mapping land degradation vulnerability in salinity- and hydrothermal-stressed regions of the southern main Ethiopian Rift

**DOI:** 10.1371/journal.pone.0359349

**Published:** 2026-09-24

**Authors:** Talema Moged Reda, Alemu Tadese, Wondimu Haimanote, Muralitharan Jothimani, Asmamaw Hangibayna, Solomon Gunta, Gizachew Kabite Wedajo, Berhan Gessesse, Admasu Adamu, Zewdneh Tomass, Guchie Gulie, Abdisa Yilma, Dereje Tsegaye

**Affiliations:** 1 Department of Remote Sensing, Space Science and Geospatial Institute, Addis Ababa, Ethiopia; 2 Department of Geology, College of Natural and Computational Sciences, Arba Minch University, Arba Minch, Ethiopia; 3 Department of Physics, College of Natural and Computational Sciences, Wolaita Sodo University, Sodo, Ethiopia; 4 Geography and Environmental Studies, Kotebe University of Education, Addis Ababa, Ethiopia; 5 Department of Environmental Science, College of Natural and Computational Sciences, Wolaita Sodo University, Sodo, Ethiopia; 6 Department of Biology, College of Natural and Computational Sciences, Wolaita Sodo University, Sodo, Ethiopia; 7 Wolaita Sodo University, Sodo, Ethiopia; 8 Space Science and Geospatial Institute, Addis Ababa, Ethiopia; 9 Department of Plant Science, College of Agricultural Sciences, Arba Minch University, Arba Minch, Ethiopia; Wallaga University, ETHIOPIA

## Abstract

Soil erosion and land degradation are the main environmental challenges disturbing ecosystem services, agricultural productivity, and community livelihoods in Ethiopia. The Southern Main Ethiopian Rift is one of the regions experiencing high levels of soil erosion and land degradation, driven by active geological processes and anthropogenic activities. However, the contribution of these geological processes to salinization and their subsequent impact on soil erosion and land degradation in the region remains poorly understood and insufficiently studied. This study was conducted to assess and map the spatial patterns of soil erosion and land degradation vulnerability in the salinized and hydrothermally stressed region of the Southern Main Ethiopian Rift using integrated multi-source earth observation data and machine learning frameworks. Landsat, Sentinel-2, SRTM DEM, CHIRPS rainfall data, soil data, and field observations were integrated with Google Earth Engine and GIS platforms to analyze existing biological, chemical, and physical indicators of land degradation. Land degradation indicators like soil erosion, land use/land cover (LULC), soil-adjusted vegetation index (SAVI), Normalized Multi-Band Drought Index (NMDI), land surface temperature (LST), salinity, and soil erosion were computed using respective approaches and integrated to produce a land degradation vulnerability map of the study area. The results show that large portions of the study area are moderately to highly vulnerable to land degradation. The major driver of land degradation in the study area is salinization, which increases surface runoff and sediment removal through surface crusting. The annual soil loss values for the area range from 25 to 100 t ha-1 yr^-1,^ with about 10.69% of the area under moderate to very severe erosion risk. Accordingly, the land degradation vulnerability map shows that 1.68%, 50.15%, 38.14%, 6.31%, and 3.72% of the study area fall into the very low, low, moderate, high, and very high vulnerability categories, respectively. The land degradation vulnerability (LDV) model demonstrated strong validation performance, with an overall accuracy of 92.34%, a Kappa coefficient of 0.87, an F1-score of 0.948, and an ROC-AUC of 0.901. These validation results demonstrate the accuracy and reliability of the LDV model for mapping land degradation vulnerability. The findings indicate that soil salinity, surface soil loss, and elevated land surface temperature, together with anthropogenic pressures, are the primary drivers of high land degradation risk in the study area.

## 1 Introduction

Land degradation describes the deterioration of land resources resulting from the combined effects of natural processes and anthropogenic activities [1,2]. It poses a significant environmental threat worldwide, severely affecting land use sustainability, ecosystem stability, agricultural productivity, and human livelihoods [3–5]. According to Wassie, 56% of degraded land globally is attributed to intense soil erosion [6]. This trend is projected to worsen, with the extent of degraded land expected to increase by 90% in 2050, potentially affecting approximately 75% of the world’s land area [7]. Land degradation predominantly occurs in arid and semi-arid regions [8]. In Africa, it is a pressing concern that negatively impacts local communities [9], reducing agricultural productivity and disrupting environmental sustainability [10]. Ethiopia is particularly vulnerable, with around 85% of its land affected by soil erosion-induced degradation [11–13]. The absence of effective soil and water conservation measures increases susceptibility to degradation [14,15]. The extent of degradation varies by region, influenced by factors such as topography [10], climate, geological material, and human activities, including urbanization, agriculture, land use changes, construction, and mining [16,17].

Land degradation occurs in three forms: biological, chemical, and physical [6]. These forms are interconnected; one can trigger another, creating a cascading effect. For example, biological degradation involves the erosion of upper soil layers, leading to a decline in organic matter and nutrient recycling [18]. Key drivers include deforestation, overgrazing, and drought, which can trigger physical or chemical degradation [19]. Signs of biological degradation include visible vegetation stress and reduced plant growth due to the loss of soil organic matter and biological activity, resulting in a decline in soil microorganisms [20–22]. Physical degradation involves the deterioration of soil properties, leading to moisture loss and reduced soil quality [23]. Common forms include waterlogging, compaction, crust formation, and erosion, which remove the upper fertile soil layer, disrupt soil structure, and cause nutrient loss, hindering root penetration and water circulation [4,24]. Disturbance of soil particles limits bulk density, water-holding capacity, and water movement, ultimately reducing crop growth and agricultural productivity [25].

Hydrothermal mineralization and alteration processes are among the chemical processes that significantly affect the physicochemical properties of soils and rocks in the rift area [26,27], changing the mineralogical composition and forming clay-rich alteration zones [28]. These processes reduce cohesion between soil grains, increasing susceptibility to weathering and promoting soil erosion and subsequently land degradation [29,30]. Salinization linked to hydrothermal fluid circulation also contributes to land degradation, particularly in areas with high evaporation rates and low precipitation [31], adversely affecting permeability, porosity, soil structure, and vegetation growth [30,32]. The degree of salinization is particularly high in arid and semi-arid regions [33]. During the high rainfall season, surface runoff and sediment transport increase due to surface crusting and reduced infiltration associated with saline soils, further reducing groundwater recharge and contributing to land degradation [33,34]. Soil salinity is a major factor in chemical degradation as excess salt limits nutrient movement by forming surface crusting [35], resulting in poor soil aeration and reduced seed germination.

Sources of salinity include natural processes and anthropogenic activities, such as high rock weathering, rising groundwater levels, seawater intrusion, chemical fertilizers, unsafe irrigation practices, and poor drainage [35]. Additionally, insufficient rainfall and high evaporation rates in arid and semi-arid regions lead to significant nutrient loss by limiting capillary rise and plants’ water uptake capacity [31,36]. On the other hand, soil acidification can also occur in areas with excess rainfall, which promotes leaching of base ions, leaving behind resistant acidic ions [37]. Important macronutrients (e.g., nitrogen, phosphorus, calcium, magnesium, potassium, sulfur) and micronutrients (e.g., iron, zinc, manganese, copper) are leached from the soil by percolating water [38,39], reducing soil fertility, altering plant growth, and affecting agricultural productivity [20,40].

Land degradation extent mapping were in done in Ethiopia where many studies have focused on anthropogenic, climatic, and topographical induced degradation [2,6,16,41,42]. For instance, Wassie, reported that 12 million hectares of land in East and South Ethiopia are affected by salinity, resulting in dry and unproductive land [6]. However, the influence of active geologic processes on salinization and their adverse impact on land degradation in the Main Ethiopian Rift system have not been sufficiently studied. This knowledge gap is important because overlooking geogenic controls such as hydrothermal alteration and salinization may lead to an incomplete characterization of degradation processes and to management interventions that primarily address climatic, topographic, or anthropogenic drivers while underrepresenting rift-specific geological controls. The methodological novelty of this study lies in integrating geogenic salinity and hydrothermal-related environmental stress with biological, moisture, thermal, land-use, and RUSLE-derived soil-erosion indicators within an integrated multi-source Earth observation, field-data, and geospatial modelling framework for land degradation vulnerability assessment in the Southern Main Ethiopian Rift. Therefore, this study aims to assess and model annual soil loss and land degradation vulnerability in hydrothermally and salinity-stressed regions of the Southern Main Ethiopian Rift by integrating multi-source Earth observation data, field observations, RUSLE-based soil-loss estimation, machine-learning-supported land-use/land-cover classification, and multi-criteria geospatial modelling.

This study contributes to land degradation research by demonstrating how geogenic salinity and hydrothermal-related environmental stress can be evaluated alongside land degradation indicators, such as land use, vegetation, moisture, thermal, and RUSLE-derived soil-erosion indicators, within an integrated land degradation vulnerability framework for an active continental rift environment. In practical terms, the framework identifies degradation hotspots that may not be adequately represented by conventional soil-erosion assessment alone, thereby providing spatial evidence for prioritizing rift-specific soil conservation, salinity management, ecosystem rehabilitation, and land-use planning.

## 2 Materials and methods

### 2.1 Description of the study area

**Fig 1:**
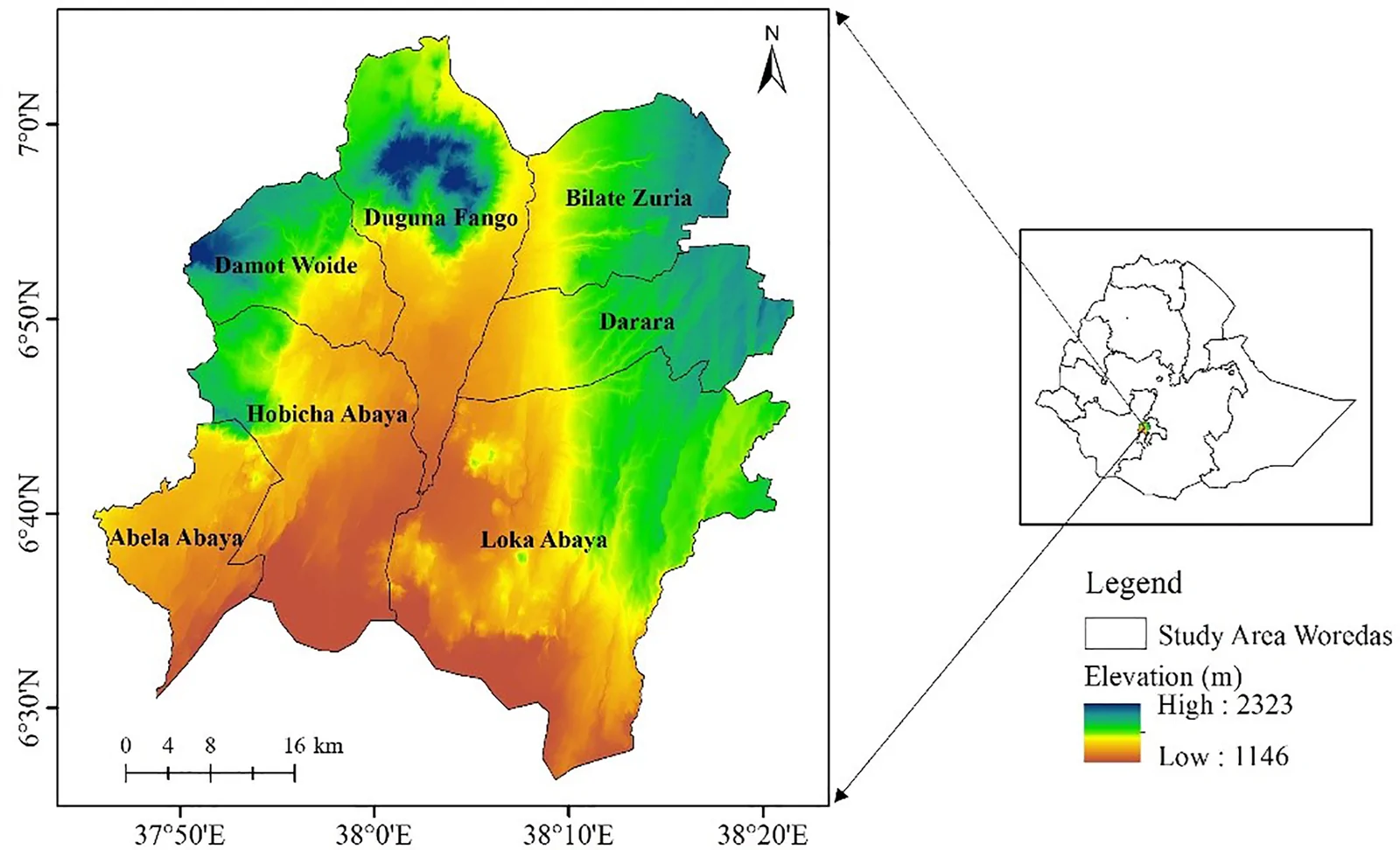
Location map of the study area.

The study area is located in the Southern Main Ethiopian Rift, comparing the two regional states of Sidamo and South Ethiopia. It is located in the geographical coordinates of 6˚30′N to 7˚0′N and 37˚50′E to 38˚20′E (Fig 1). The area has flat and undulating topography, with elevations ranging from 1146 m to 2323 m above sea level (masl). It is part of the East African Rift System, which is dominated by active volcanic-tectonic processes and complex geological and geomorphological conditions. The area plays a significant role in scientific investigations of crustal deformation, magmatic processes, geohazards, geothermal and mineral resources, and environmental studies. The outcrop of the area is covered by pyroclastic materials (ignimbrite, unwelded tuff, and pumice) and dark-colored vesicular basalt. These rock units are mainly exposed along hillsides, rivers, and road cuts. There is also a travertine deposit indicating past hydrothermal activity. The travertine displays clear zoning with a structural alignment in the S20^0^W or N20^0^E direction. Deep gully erosion has formed in the thick soil deposit. The topsoil layer is white, indicating salinity resulting from precipitation derived from elevated evaporation. The region has a bimodal rainfall pattern, with minimum rainfall (Belg) from March to May and high rainfall (Kiremt) from June to August.

### 2.2 Data type and their sources

In this study, Earth observation satellite data, global ancillary datasets, and field observations were integrated to model land degradation vulnerability. Sentinel-2 high-resolution multispectral data for 2025 were obtained from the European Space Agency (ESA) Copernicus Program using the Google Earth Engine (GEE) Data Catalogue. The Sentinel-2 Multi-Spectral Instrument (MSI) provides consistent high-resolution imaging of the Earth’s surface over a wide swath, with a 5-day revisit interval and spatial resolutions of 10 m, 20 m, 20 m, and 60 m across 13 spectral bands. The spectral resolution spans a broad range of the electromagnetic spectrum, including nine visible and near-infrared bands with wavelengths from 0.433 to 0.748 μm, five near-infrared bands with wavelengths from 0.773 to 1.39 μm, and two shortwave infrared bands with wavelengths from 1.565 to 2.28 μm. In this study, various surface reflectance bands are used for Land Use Land Cover (LULC) classification and to generate vegetation condition indices, drought indicator indices, and soil salinity indices.

Landsat 8 Thermal infrared data were obtained from the GEE Data Catalogue from the USGS Landsat program. The thermal bands (specifically Band 10, ST_B10) were collected from atmospheric-corrected, land-surface emissivity-adjusted Level-2 collections for 2025. These thermal bands were used to calculate the mean annual Land Surface Temperature (LST) of 2025.

Gridded, high-resolution Climate Hazards Group InfraRed Precipitation with Station (CHIRPS) precipitation data was acquired from the GEE Data Catalogue, released by the University of California, Santa Barbara (UCSB). CHIRPS integrate the long-term satellite estimates with in-situ station data. In this study, CHIRPS was used to estimate mean annual rainfall and the predictive rainfall erosivity factor, which supports the estimation of potential rainfall-induced surface runoff and soil stripping during high-intensity wet seasons. Further, the Shuttle Radar Topography Mission (SRTM) dataset was obtained from the GEE Platform and used to extract terrain parameters, including elevation, slope steepness, and slope length.

Soil type data were obtained from global soil databases managed by the Food and Agriculture Organization (FAO). This dataset consists of site-specific spatial thematic maps representing regional soil types and textural arrangements. In this study, soil type data were used to estimate soil erodibility factor for the Revised Universal Soil Loss Equation (RUSLE) soil loss estimation model. The datasets used in this study are summarized in Table 1. Field surveys and expert judgments in the targeted active rift valley Woredas were used to develop the primary validation dataset. No formal fieldwork permit was required because the field activities were limited to non-invasive environmental observations and GPS-based ground-control-point collection for land-use/land-cover classification and model validation, without collection or removal of biological, geological, or other physical specimens.

**Table 1:** Summary of data sources and their purposes.

Degradation Pathway	Indicator	Source Organization	Purpose
Biological	LULC	ESA / GEE Data Catalog	Executing supervised LULC classification using machine learning.
	SAVI	ESA / GEE Data Catalog	Quantifying the greenness and stress of vegetation conditions in sparse or exposed soil environments.
	NMDI	ESA / GEE Data Catalog	Estimating soil moisture and canopy water stress to delineate drought-susceptible zones.
	LST	USGS / GEE Data Catalog	Assessing surface thermal impacts to identify hyper-arid and geothermal degradation hotspots.
Hydrogeochemical	Soil Salinity	ESA / GEE Data Catalog	Inverting and mapping regional soil salinity and surface crusting across hydrothermal fluid zones.
Physical (Soil Erosion)	Slope & Slope Length Factor	NASA / GEE Data Catalog	Extracting slope steepness, and catchment flow parameters to simulate erosion vulnerability.
	Rainfall Erosivity Factor	UCSB Climate Hazards Group / GEE	Computing annual precipitation to assess water-driven runoff and topsoil detachment energy.
	Soil Erodibility Factor	FAO Global Database	Determining the inherent susceptibility of localized soil profiles to mechanical detachment and transport.
Validation & Training	Model validation & Accuracy Assessment	Hand-held GPS Field observation & High-Res Imagery	Providing robust training samples (70%) and testing records (30%) for image classification and machine learning performance validation.

To resolve spatial resolution mismatches among multi-source datasets (ranging from 10 m to ~5 km), a spatial harmonization protocol was implemented in Google Earth Engine (GEE) before multi-criteria integration. All spatial layers were reprojected to a unified coordinate system (WGS 1984 datum) and snapped to a consistent analysis grid. Coarser continuous variables, specifically CHIRPS precipitation (~5 km) and thermal LST, were resampled using bilinear interpolation to construct smooth, continuous spatial gradients suitable for macro-climatic factors such as rainfall erosivity (R). Conversely, high-resolution optical features (Sentinel-2) and discrete categorical inputs (FAO soil classes) were resampled using nearest-neighbor interpolation to preserve distinct spatial boundaries and prevent pixel-value distortion.

### 2.3 Methods

#### 2.3.1 LULC classification.

The LULC was classified from Sentinel-2 MSI surface reflectance datasets for 2025 obtained from the GEE data catalogue (“COPERNICUS/S2_SR_HARMONIZED”). First, a cloud-filtering technique was applied to select pixels with cloud coverage <20%. Accordingly, cloud cover and shadow images were removed using specific bit masks: cloudBitMask = 1 << 10 and cirrusBitMask = 1 << 11. A cloud-free composite image was generated by taking the median value of a collection of satellite images, which was then clipped to the area of interest on the GEE computing platform. For the LULC classification, five Sentinel-2 MSI surface reflectance bands (B2, B3, B4, B8, B11, and B12) were used.

Furthermore, terrain variables (such as elevation and slope), vegetation condition indices like Enhanced Vegetation Index (EVI), Normalized Difference Vegetation Index (NDVI), Bare Soil Index (BSI), Normalized Difference Built-up Index (NDBI), and Normalized Difference Water Index (NDWI) were used to enhance the accuracy of LULC classification [43–45]. A total of 4,797 Ground Control Points (GCPs) were collected through three rounds of targeted field surveys conducted in January, February, and March 2025 using handheld GPS receivers (positional accuracy of **±**5m), supplemented by high-resolution Google Earth Pro imagery to verify inaccessible areas. The GCPs were gathered across various LULC types: bare land (733), built-up areas (621), cropland (1,341), forest land (334), grassland (847), shrubland (721), water bodies (75), and wetlands (125), as outlined by [44]. Field validation protocols included cross-verifying each point with local knowledge, using mean annual imagery to confirm seasonality, and removing edge/boundary pixels to minimize spectral mixing. Of the collected data, 70% was used to train the model, while 30% was reserved for validation via stratified random sampling, as described by Sakti et al. (2024). A random forest machine learning image classification algorithm with 100 trees was used for LULC classification on GEE. The accuracy of LULC classification was evaluated using precision, recall, F1-score, overall accuracy, and kappa coefficient, as reported by [43]. The formulas described by [43] were used to compute the statistical evaluation metrics for the confusion matrix. Based on an extensive literature review and expert insights, the LULC of the study area was reclassified into five sub-classes of land degradation vulnerability (Table 9).

#### 2.3.2 Soil adjusted vegetation index (SAVI).

Vegetation cover serves as an important biological indicator for assessing land degradation vulnerability. This study employed SAVI because it performs better than NDVI in areas with sparse vegetation and exposed soils by minimizing the influence of soil background reflectance [46,47]. It is used to assess vegetation greenness. Using the GEE computational platform and JavaScript code, the mean annual SAVI of 2025 was calculated from the near-infrared (NIR) and Red(R) bands of Sentinel-2 MSI data. The SAVI values range from −1 (stressed vegetation) to 1 (healthy vegetation). It was calculated using Equation (1) as described by [46]:


$$\begin{array}{c} SAVI=\frac{\left(NIR-R\right)}{\left(NIR+R+L\right)}(\text{1}+L) \end{array}$$ (1)

where NIR represents the surface reflectance of the near-infrared band, R denotes the surface reflectance of the red band, and L is the soil brightness correction factor of 0.5, as stated by [46]. Then the SAVI thematic layer was reclassified into five sub-classes based on land degradation vulnerability (Table 9).

#### 2.3.3 Normalized multi-band drought index (NMDI).

NMDI is a newly developed remote sensing drought index for assessing soil and vegetation moisture stress [48]. This index highlights the limitations of other vegetation indices, especially the significant influence of soil on reflectance values [49,50]. In this study, NMDI is used to assess the stress imposed by soil moisture and drought conditions in the study area. The mean annual NMDI of 2025 was calculated from Sentinel-2 multispectral surface reflectance bands of NIR and Short-Wave Infrared (SWIR 1 and 2) using Equation (2) as described by [48] through the GEE computational platform and JavaScript code. The NMDI values range from −1 (dry condition) to 1 (wet condition).


$$\begin{array}{c} NMDI=\frac{\left(NIR-\left({SWIR}_{\text{1}}-{SWIR}_{\text{2}}\right)\right)}{\left(NIR+\left({SWIR}_{\text{1}}-{SWIR}_{\text{2}}\right)\right)} \end{array}$$ (2)

where NIR denotes the surface reflectance of the near-infrared band, while ${SWIR}_{\text{1}}$ and ${SWIR}_{\text{2}}$ represent the surface reflectance of shortwave infrared 1 and 2, respectively.

#### 2.3.4 Mean annual LST estimation.

LST is also an essential indicator for assessing land degradation vulnerability [5]. The mean annual LST of the study area was computed from atmospherically corrected, land-surface emissivity-adjusted Landsat 8 Level 2 data for 2025 using the ST_B10 band. A cloud mask of less than 20% was applied to compute the mean LST in degrees Celsius (°C) on the GEE platform using equation (3).


$$\begin{array}{c} LST{(}^{\circ }\text{C})=(ST*\text{0}.\text{00341802}+\text{149})-\text{2}\text{7}\text{3}.\text{1}\text{5} \end{array}$$ (3)

where ST represents the surface temperature of ST_B10 (Landsat 8). The LST thematic layer was reclassified into five sub-classes of land degradation vulnerability (Table 9).

#### 2.3.5 Soil salinity mapping.

Volcanically and tectonically active regions are characterized by elevated heat flow, closely spaced faulting, and widespread hydrothermal circulation. In these geological settings, meteoric waters can infiltrate along fault zones and interact with molten materials at depth, re-emerging as thermal springs at the surface, enriched with dissolved ions such as Na⁺ , Ca^2+^, HCO_3_^_-_^, and F^-^ . During discharge, processes including CO_2_ degassing, evaporation, and mineral super saturation can promote the precipitation of carbonate salts and travertine, leading to localized soil salinity, sodicity, and alkalinity. Soil salinization or the accumulation of soluble salts (chlorides and sulfates) in the soil, results from both geogenic processes and human activities [51]. The processes of salinization can bind soil particles and disrupt soil structure, resulting in poor soil structure, surface crusting, drainage issues, and reduced permeability, thereby reducing water infiltration. Therefore, salinization represents a significant form of land degradation in hydrothermal fluid environments of arid and semi-arid regions.

In this study, various Sentinel-2 MSI spectral indices such as the salinity index, salinity index red edge i, salinity index 1, salinity index 2, salinity index 3, salinity index 1 red edge i, salinity index 2 red edge i, salinity index 3 red edge i, salinity index I, salinity index II, salinity index III, intensity index 1, intensity index 2, and salinity index-11 were used to map the spatial distribution of soil salinity as explained by [51–53]. After an intensive analysis of these spectral indices, Intensity Index 2 (Int2) was used to assess land degradation vulnerability, as it effectively demonstrated the ability to detect salinized regions of the study area. Further, the salinization intensity index was reclassified into five classes based on their relevance for land degradation vulnerability mapping. Soil salinity indices were calculated using the mathematical equations as presented in Table 2.

**Table 2:** Soil salinity spectral indices calculation.

Salinity indices	Formula	References
Salinity index (SI)	$\sqrt{B*R}$	[51,54]
Salinity index red edge i (SIrei)	$\sqrt{B*Rei}$	[51,55]
Salinity index 1 (SI1)	$\sqrt{G*R}$	[51,56]
Salinity index 2 (SI2)	$\sqrt{G^{\text{2}}+R^{\text{2}}+NIR^{\text{2}}}$	
Salinity index 3 (SI3)	$\sqrt{G^{\text{2}}+R^{\text{2}}}$	
Salinity index 1 red edge i (SI1rei)	$\sqrt{G*Rei}$	[51,55]
Salinity index 2 red edge i (SI2rei)	$\sqrt{G^{\text{2}}+{Rei}^{\text{2}}+{NIR}^{\text{2}}}$	
Salinity index 3 red edge i (SI3rei)	$\sqrt{G^{\text{2}}+{Rei}^{\text{2}}}$	
Salinity index I (S1)	B/R	
Salinity index II (S2)	(B-R)/(B + R)	
Salinity index III (S3)	(G*R)/B	
Intensity index 1(Int1)	(G + R)/2	
Intensity index 2 (Int2)	(G + R + NIR)/2	
Salinity index-11 (SI-11)	(SWIR1*SWIR2)	[51,57]

#### 2.3.6 Soil loss estimation using the RUSLE model.

Soil loss is an important physical indicator of land degradation for modeling land degradation vulnerability, helping identify highly vulnerable areas to soil erosion. The model incorporates the Rainfall Erosivity Factor (R), Soil Erodibility Factor (K), Slope Length and Steepness Factor (LS), Cover Management Factor (C), and Support Practices Factor (P) to quantify annual soil loss in the study area.

a. **Rainfall Erosivity Factor (R)**

The rainfall erosivity factor (R) quantifies the kinetic energy and intensity of raindrop that cause soil detachment and transportation [58]. In data-scarce regions lacking high-frequency pluviographical stations, regional empirical models calibrated to local climate conditions are widely applied. For Ethiopian climatological conditions, R was computed using the empirical relationship established under the Soil Conservation Research Programme and widely adapted across Ethiopian rift and highland catchments [10]:


$$\begin{array}{c} R=(\text{0}.\text{5}\text{6}\text{2}\times P)-\text{8}.\text{1}\text{2} \end{array}$$ (4)

where R is the rainfall erosivity factor (MJ mm ha − 1 h − 1 year−1), and P is the mean annual rainfall (mm/year).

b. **Soil Erodibility Factor (K)**

The K factor is used to assess soil erosion vulnerability across various soil types based on soil physicochemical properties such as texture, structure, and permeability [58,59]. For this study, the K factor was obtained from the FAO soil map; it ranges from 0.11 (low vulnerability to erosion) to 0.33 (high vulnerability to erosion) (Table 3).

**Table 3:** K-factor values for various soil types based on existing literature.

No	Soil types	K_values	References
1	Calcaric Flubisols, Calcic Fluvisols, Chromic Vertisols, and Vitric Andosols	0.2	[60–63], Expert Judgment
2	Eutric Nitisols, Pellic Vertisols,	0.15	
3	Dystric Nitisols, Leptosols	0.25	
4	Chromic Luvisols	0.11	
5	Eutric Fluvisols	0.18	
6	Eutric Regosols	0.33

c. **Slope Length and Steepness Factor (LS)**

Topography is one of the most important factors used to quantify soil loss, including slope length and steepness [10,58]. Slope length and steepness affect surface runoff. The steeper and longer slopes are more vulnerable to soil erosion, whereas the shorter, flatter slopes are less vulnerable. The LS factor was extracted from Shuttle Radar Topography Mission (SRTM) elevation data with a 30 m spatial resolution. LS factor was computed from flow accumulation and slope using equation (5) on GEE, as outlined by [58].


$$\begin{array}{c} LS={\frac{(FlowAccumulation\times cellsize}{\text{2}\text{2}.\text{1}\text{3})}}^{\text{0}.\text{4}}\times {\frac{\left(\text{sin}(Slope\times \text{0}.\text{0}\text{1}\text{7}\text{4}\text{5}\right)}{\text{0}.\text{0}\text{8}\text{9}\text{6})}}^{\text{1}.\text{3}} \end{array}$$ (5)

where LS refers to the slope length and steepness factor; flow accumulation is extracted from the flow direction of the filled DEM data at a spatial resolution (cell size) of 30 m; and slope is the steepness of the area in degrees.

d. **Cover Management Factor (C)**

The cover management factor in the RUSLE model indicates the influence of agricultural practices, cropping systems, vegetation cover, surface roughness, and land management on soil erosion [64]. The C values were derived from the LULC types to assess soil erosion susceptibility [10,60,65]. In this study, water bodies were masked out from the C factor calculation to avoid misinterpretation of soil erosion, and the C factor was linked to land management practices in terrestrial environments. The C values range from 0 to 1, with values near 0 indicating strong land management practices and a greater area covered by vegetation and forests. In contrast, values near 1 indicate poor land management practices and a greater area covered by bare land [10]. The C values for different LULC types are presented in Table 4.

**Table 4:** C-factor values for different LULC types based on existing literature.

No	LULC types	C-values	References
1	Bare land	0.6	[10,44,59,60,65]
2	Built-up	0.1	
3	Cropland	0.25	
4	Forest	0.001	
5	Grassland	0.05	
6	Shrub land	0.03	
7	Wetland	0. 01

e. **Support Practices Factor (P)**

The P factor assesses the effectiveness of soil conservation practices, such as strip cropping, strip tillage, and contouring, in minimizing soil erosion. The P values range from 0 to 1, with values near 0 indicating proper soil conservation practices and those near 1 indicating poor soil conservation practices [10,60,63,66]. The P factor was derived by considering LULC classes and slope of the area. Table 5 presents the P values for different LULC types and slope classes.

**Table 5:** P-factor values for different LULC and slope classes based on existing literature.

No	LULC	Slope (%)	P value	References
1	Cropland	0-5	0.1	[4,10]
		5-10	0.12	
		10-20	0.14	
		20-30	0.19	
		30-50	0.25	
		>50	0.33	
2	Bare land	All slope	0.73	[60]
3	Built-up	All slope	0.63	
4	Forest land	All slope	0.53	
5	Grassland	All slope	0.63	
6	Shrub land	All slope	0.6	[63]
7	Wetland	All slope	0.7	[66]

The GEE platform facilitates comprehensive analysis of annual soil loss and its driving factors by integrating multiple datasets from various sources and efficiently processing large volumes of data. The RUSLE model was performed on the GEE platform using multi-source remote sensing data to estimate mean annual soil loss. Rainfall, soil type, digital elevation model, and LULC were used to calculate the R, K, LS, C, and P factors, respectively. The final annual soil loss thematic layer was computed by multiplying each factor map using Equation (6), as described by [58].


$$\begin{array}{c} A=R\times K\times LS\times C\times P \end{array}$$ (6)

where A is the annual soil loss in tons per hectare per year, R is the rainfall erosivity factor, K is the soil erodibility factor, LS is the slope length and steepness, C is the cover and management factor and, P is the conservation practice.

**Fig 2:**
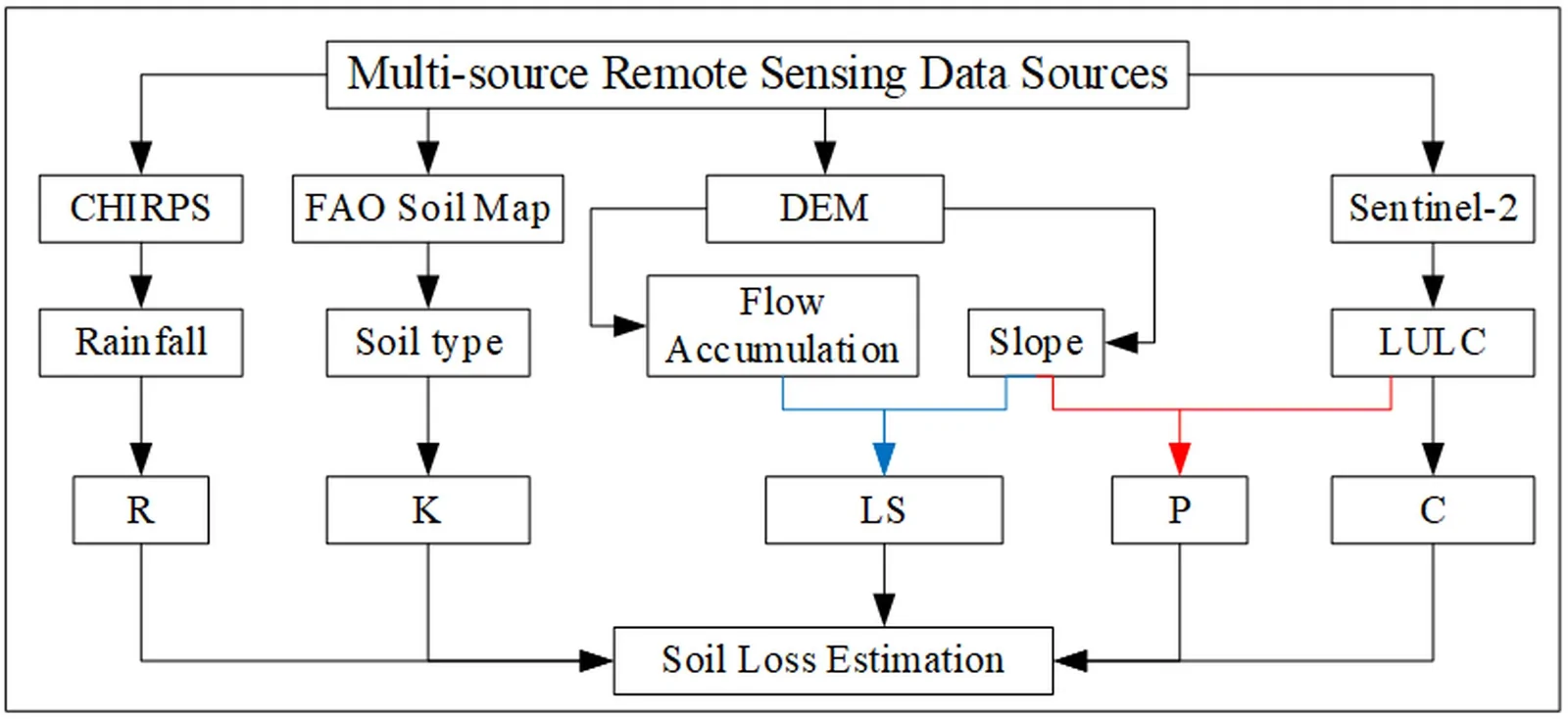
Methodological flow chart for mean annual soil loss estimation.

The final soil loss thematic map was used as input for land degradation vulnerability mapping. The general methodological flow chart for estimating mean annual soil loss is shown in Fig 2.

#### 2.3.7 Multi-criteria decision analysis (MCDA).

Saaty introduced the Analytic Hierarchy Process (AHP), a multi-criteria decision-making (MCDM) methodology [67]. Sandeep et al. then combined AHP-MCDA with GIS-based modeling to evaluate land degradation [68]. The study utilized the AHP to integrate six key indicators of land degradation: LULC, SAVI, NMDI, LST, salinity, and soil loss within a multi-criteria decision analysis framework. In the MCDA framework, a pairwise comparison matrix was used to assign weights to interdependent land degradation vulnerability (LDV) indicators based on their relative importance (Table 6). To ensure spatial consistency in the LDV study, all indicators were standardized to the WGS 1984 coordinate system and resampled to a uniform spatial resolution of 0.0027 degrees (30m) using bilinear interpolation. The resampled thematic layers were then reclassified and standardized to a common land degradation vulnerability score ranging from very low (1) to very high (5). Class-level scores and weights for each indicator were established based on expert judgment, local climate and environmental conditions, and evidence from previous studies conducted in semi-arid and arid environments. Table 6 shows the pairwise comparison matrix and weights for six indicators of land degradation vulnerability.

**Table 6:** Pairwise comparison matrix for quantifying land degradation vulnerability.

Indicator	LULC	SAVI	NMDI	LST	Salinity	Soil loss
LULC	1	1/2	1	1	1/5	1/5
SAVI	2	1	2	2	¼	1/3
NMDI	1	1/2	1	2	1/3	1/3
LST	1	1/2	1/2	1	1/5	1/5
Salinity	5	4	3	5	1	½
Soil loss	5	3	3	5	2	1
Column Sum	15	9.5	10.5	16	3.98	2.57

In the Analytic Hierarchy Process (AHP), the column sums for each indicator are obtained by summing all values in each column (Table 6). The normalized weighted matrix was obtained by dividing each column value by its column sum (Table 8). Subsequently, the weight of each indicator was calculated by averaging the values in each row of the normalized matrix. Consistency Index (CI) and Consistency Ratio (CR) are utilized to evaluate the accuracy of the Analytic Hierarchy Process (AHP) pairwise comparison matrix. In the normalized weight matrix, the Principal Eigenvalue ($\lambda_{max}$), CI, and CR were calculated using equations (7), (8), and (9), respectively, as described by [68].


$$\begin{array}{c} \lambda_{max}=\left(\sum_{i=\text{1}}^{n}\lambda_{i}\right)/n \end{array}$$ (7)

$$\begin{array}{c} CI=\frac{\lambda_{max}\;-n}{n-\text{1}} \end{array}$$ (8)

$$\begin{array}{c} CR=\frac{CI}{RI} \end{array}$$ (9)

Where n and RI represent the number of LDV indicators (n = 6) and the random index (RI = 1.24), respectively (Table 7).

**Table 7:** Random Index (Saaty, 1980).

n	1	2	3	4	5	6	7	8	9	10
RI	0.00	0.00	0.58	0.90	1.12	1.24	1.32	1.41	1.45	1.49

In the normalized weighted matrix shown in Table 8, the computed values of λ_max, CI, and CR were 6.16, 0.032, and 0.026, respectively. The CR result demonstrated the reliability of the normalized weight matrix, which was below the acceptable threshold of 0.1 [67,68]. Accordingly, soil and salinity have the highest weights, indicating they are the most important factors compared to the others.

**Table 8:** Normalized weighted matrix for land degradation vulnerability indicators.

Indicator	LULC	SAVI	NMDI	LST	Salinity	Soil loss	Row mean	Weight (%)	$\lambda_{𝐢}$
LULC	0.07	0.05	0.1	0.06	0.05	0.08	0.07	6.8	6.14
SAVI	0.13	0.11	0.19	0.13	0.06	0.13	0.12	12.4	6.2
NMDI	0.07	0.05	0.1	0.13	0.08	0.13	0.09	9.3	6.13
LST	0.07	0.05	0.05	0.06	0.05	0.08	0.06	6	6.11
Salinity	0.33	0.42	0.29	0.31	0.25	0.2	0.3	30	6.18
Soil loss	0.33	0.32	0.29	0.31	0.5	0.39	0.36	35.7	6.22
λ_max									**6.16**

The land degradation vulnerability map was generated through a weighted linear combination of six indicators, as indicated in equation (10) and Table 9.

**Table 9:** Indicators, sub-indicators, class values, scores, and weights for LDV mapping.

Indicators	Sub-indicators	Class value	Scoring	Weight	Reference
Biological Indicator (35%)	LULC	Bare land	5	6.8%	[27,69]
		Built-up	4		
		Cropland	3		
		Forest	1		
		Grassland	3		
		Shrub land	2		
		Waterbody	Mask out		
		Wetland	2		
	SAVI	<0.15	5	12.4%	[69]
		0.15-0.2	4		
		0.21-0.3	3		
		0.31-0.4	2		
		>0.4	1		
	NMDI	<0.5	1	9.3%	[48]
		0.5-0.55	2		
		0.56-0.6	3		
		0.61-0.7	4		
		>0.7	5		
	LST	<30	1	6%	[70]
		30-35	2		
		35.1-40	3		
		40.1-45	4		
		>45	5		
Chemical Indicator	Salinity	<0.19	1	30%	[51,55]
		0.19-0.23	2		
		0.231-0.26	3		
		0.261-0.3	4		
		>0.3	5		
Physical Indicator	Soil loss estimation (RUSLE)	<25	1	35.7%	[69]
		25-50	2		
		50.1-75	3		
		75.1-100	4		
		>100	5		


$$\begin{array}{c} LDV=(LULC\times 0.068)+(SAVI\times 0.124)+(NMDI\times 0.93)+(LST\times 0.06)+(Salinity\times 0.3)+(Soil\text{\textasciitilde{}}loss\times 0.357) \end{array}$$ (10)

#### 2.3.8 Validation analysis.

Validation of model performance is crucial to ensure the reliability, accuracy, and applicability of the final Land Degradation Vulnerability (LDV) map [71–73]. To validate the LDV map, a proportionate stratified random sampling strategy was employed, yielding 640 ground control points (GCPs) collected through field observations and integrating high-resolution satellite imagery. This sample size exceeds standard statistical sampling recommendations for regional accuracy assessment [74–76], which mandate a minimum of 50–100 reference points per class to achieve a 95% confidence level with a margin of error of less than 5% across landscape scales.

To avoid spatial prevalence bias and accurately reflect real-world landscape proportions [77], the GCP distribution was stratified proportionally to the areal extent of each class across the 2,635 km^2^ study area, resulting in 230 points in vulnerable areas (~36%) and 410 points in non-vulnerable areas (~64%). The LDV values at each ground reference point were extracted using the Extract Multi Values to Points tool in ArcGIS to verify the final map outputs.

Statistical computations were executed in R using the pROC and caret packages to compute Receiver Operating Characteristic (ROC) curves, Area Under the Curve (AUC), overall accuracy, and Kappa coefficients. The ROC curve evaluates model performance by plotting the true positive rate against the false positive rate. AUC values range from 0 to 1, with higher values indicating better discrimination. Following Torabi Haghighi et al, AUC values were classified into five performance levels: poor (0.5–0.6), fair (0.6–0.7), good (0.7–0.8), very good (0.8–0.9), and excellent (0.9–1.0) [73]. Similarly, Kappa coefficients were categorized to evaluate agreement: poor (<0.4), fair (0.4–0.55), good (0.55–0.85), very good (0.85–0.99), and perfect (0.99–1.0) [73].

#### 2.3.9 Spearman’s correlation analysis.

**Fig 3:**
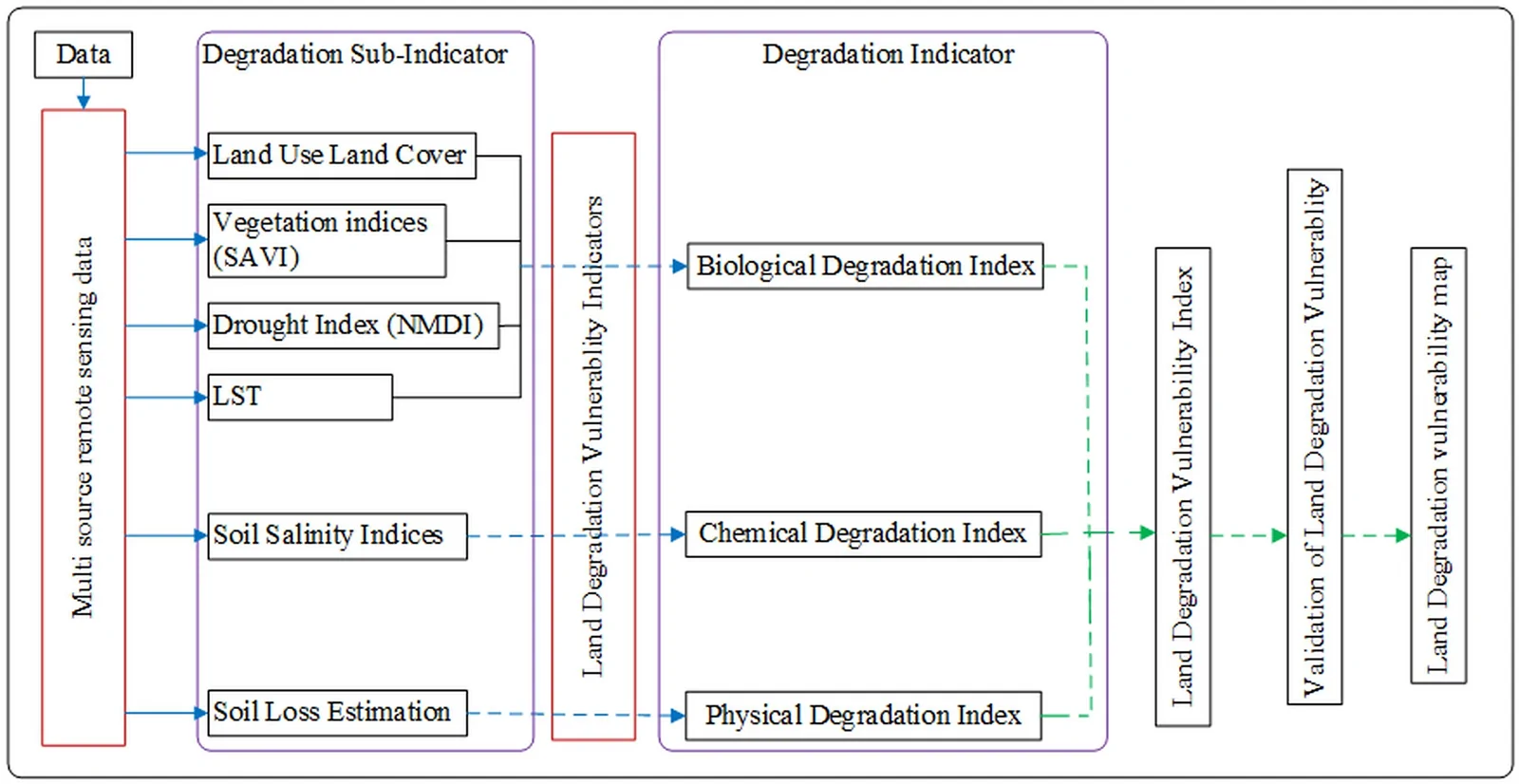
General methodological flow chart for land degradation vulnerability.

The Spearman’s correlation coefficient (ρ) was employed to assess the pairwise relationship between LULC, SAVI, LST, NDMI, Soil loss, Salinity, and LDV. This coefficient helps determine the strength and direction of monotonic relationships in ordinal data. All land degradation indicators and the final LDV map were reclassified into 5 classes: very low (1), low (2), moderate (3), high (4), and very high (5). The R corrplot and stats packages were used to visualize the pairwise relationship between the land degradation indicator and LDV using a correlation heatmap. The ρ value ranges from −1–1, where a value of ρ closer to 1 indicates a strong positive association between two variables. In contrast, a value of ρ closer to −1 indicates a weak negative association between two variables. Based on Ali Abd Al-Hameed, the ρ values are grouped into 4 association classes: strong (0.7 < |ρ| ⩽ 0.99), moderate (0.5 < |ρ| ⩽ 0.69), low (0.3 < |ρ| ⩽ 0.49), and weak (0.01 < |ρ| ⩽ 0.29) [78,79]. Fig 3 illustrates the general methodological framework employed in this study.

## 3 Results

### 3.1 LULC classification

The overall accuracy and Kappa coefficient of the land use/land cover classification are 87.82% and 0.85, respectively, both within the acceptable range. The LULC map indicates that cropland and grassland were the dominant LULC classes, covering 32.80% (879.39 km2) and 28.17% (755.50 km2) of the study area in 2025, respectively, suggesting the presence of intensive agricultural practices and semi-natural vegetation (Table 10). Shrub land accounted for 23.70% (635.16 km^2^) coverage, whereas forest land (4.79%, 128.33 km^2^), Waterbody (1.69%, 45.32 km^2^), and Wetland (1.40%, 37.58 km^2^) exhibited relatively small coverage across the total 2,680.77 km^2^ landscape. The limited extent of forest land is primarily due to historical and ongoing agricultural expansion into natural vegetation, as well as to environmental conditions that favour shrubland and grassland succession. The result demonstrates the potential ecological pressures and land-use changes in the area.

**Table 10:** LULC accuracy assessment and area coverage.

LULC	Accuracy Assessment (%)			Area Coverage	
	Precision (user accuracy)	Recall (producer accuracy)	F1-score	Area (km^2^)	Area (%)
Bare land	89.19	90	89.59	191.9	7.16
Built-up	98.37	97.31	97.83	7.59	0.29
Crop land	85.41	90.29	87.78	879.39	32.8
Forest land	93.48	86	89.58	128.33	4.79
Grass land	82.17	74.4	78.09	755.5	28.17
Shrub land	84.14	88.42	86.23	635.16	23.7
Waterbody	91.67	100	95.65	45.32	1.69
Wetland	96.97	86.49	91.42	37.58	1.4
Overall Accuracy (%)	87.82				
Kappa Coefficient	0.85				
Total				2680.77	100

**Fig 4:**
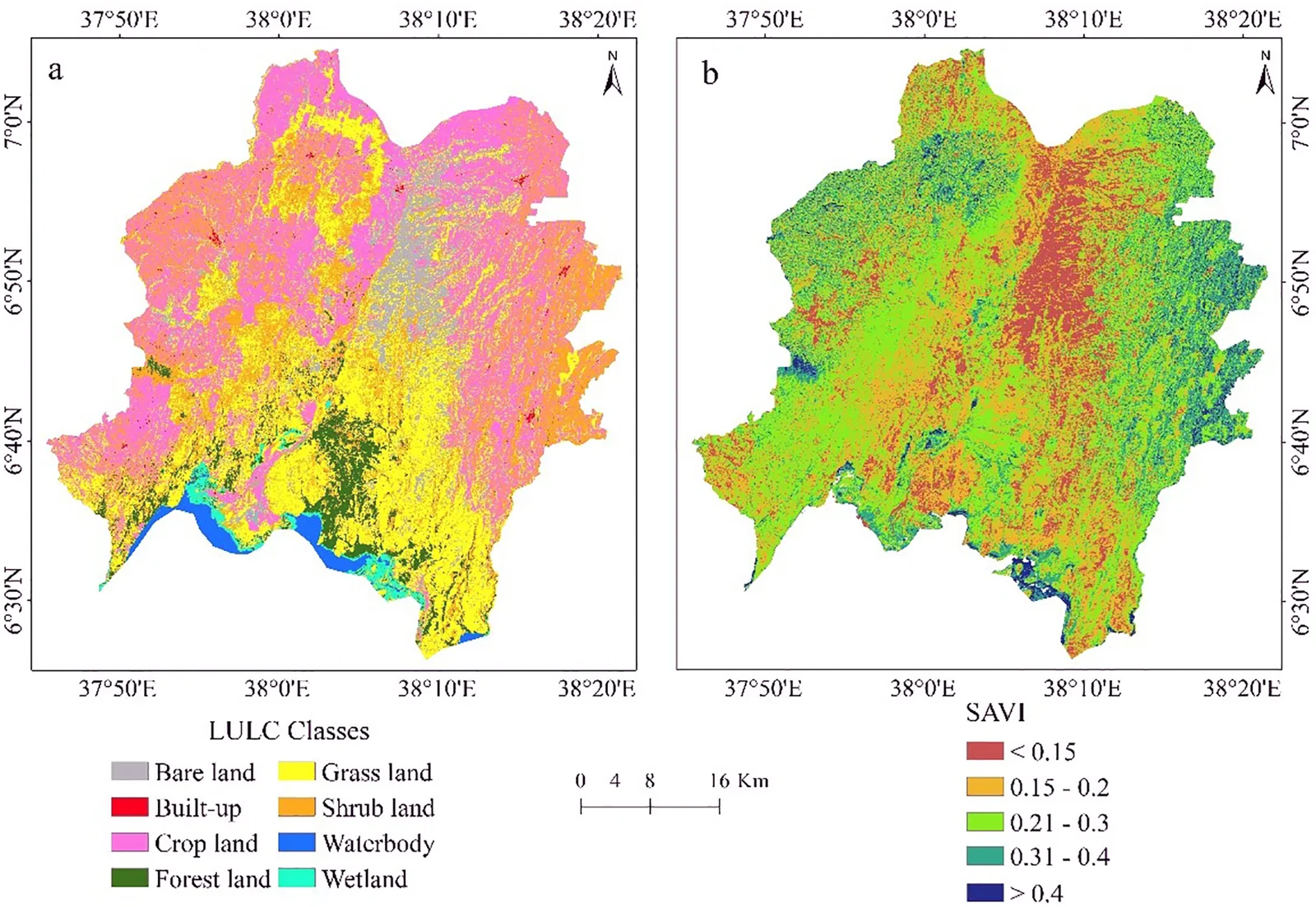
LULC map (a), SAVI map (b).

Table 11 shows LULC classes, areal extent, and land degradation vulnerability levels. Accordingly, bare land, which covers 7.28% of the total study area, is categorized as very highly vulnerable to land degradation due to severe soil erosion and the absence of vegetation. The larger proportion of the area, which is covered by cropland and grassland, was categorized as moderately vulnerable to land degradation (62.03%). The remaining LULC classes, such as shrubland and wetlands, were categorized as having low vulnerability to degradation (25.53%). Forest and water bodies (4.87%) were categorized as areas with very low vulnerability to degradation. Therefore, the LULC analysis reveals that the study area is experiencing severe land degradation, particularly in agricultural, built-up, and bare land areas (Table 11, Fig 4a). Such land degradation could result from the combined effects of unrestricted human activities and a geodynamic event along the rift segment.

**Table 11:** LULC classification in the study area.

Classes	Score	Land degradation vulnerability level	Area (km^2^)	Area (%)
Forest	1	Very low	128.33	4.87
Shrubland, Wetland	2	Low	672.69	25.53
Cropland, Grassland	3	Moderate	1634.8	62.03
Built-up	4	High	7.59	0.29
Bare land	5	Very high	191.9	7.28

### 3.2 Vegetation indicators (SAVI)

The Soil Adjusted Vegetation Index (SAVI) analysis results clearly show that the study area is affected by a high degree of land degradation, with the extent varying spatially. SAVI shows areas with less vegetation and greater exposure to surface soil erosion. The dominant portion of the area falls under moderate to high levels of land degradation vulnerability (Table 12, Fig 4b).

**Table 12:** Soil adjusted vegetation index (SAVI).

Classes	Score	Land degradation vulnerability level	Area (km^2^)	Area (%)
<0.15	5	Very high	381.23	14.47
0.15-0.2	4	High	678.05	25.73
0.21-0.3	3	Moderate	1089.6	41.34
0.31-0.4	2	Low	407.15	15.45
>0.4	1	Very low	79.42	3.01
Total			2635.45	100

Areas with Soil Adjusted Vegetation Index values ranging from 0.21 to 0.30indicate moderately degraded land (41.34%) (Table 12). Likewise, 25.73% and 14.47% of the area is in high and very high vulnerability to vegetation cover degradation, respectively. Areas with low SAVI are associated with cultivated and bare lands that are susceptible to erosion and indicate human-modified landscapes and poor land management. The remaining 18.46% of the area with a high SAVI value (>0.31) is characterized by low to very low levels of land degradation. They are relatively good vegetation coverage, indicating forest lands, wetlands, and some protected lands. Based on the SAVI analysis, more than 80% of the study area is at a moderate to very high level of land degradation risk. Therefore, it is alarming that ecological stress exists and that there is an urgent need for safe and appropriate sustainable land management interventions.

### 3.3 Drought indicator (NMDI)

**Fig 5:**
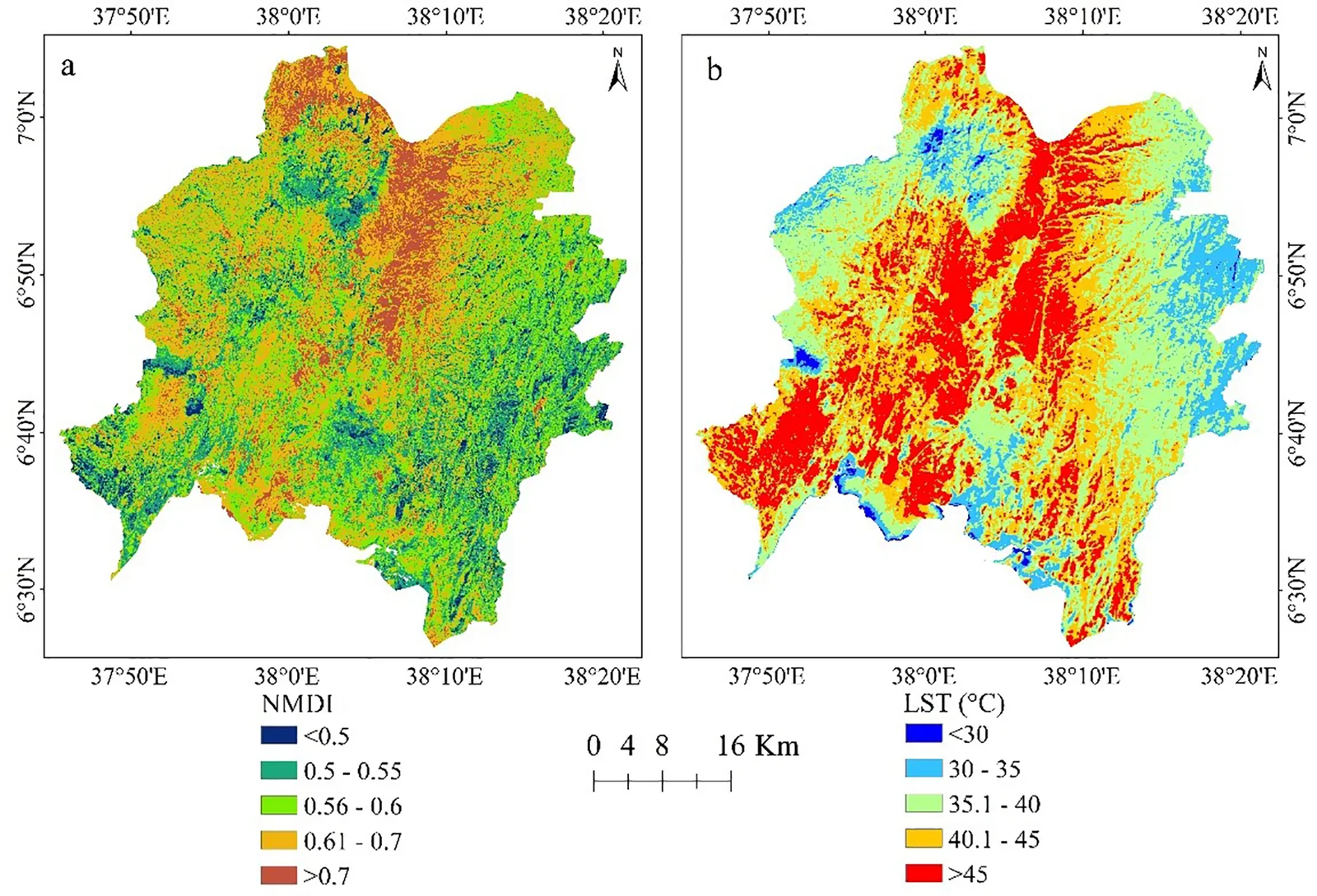
NMDI map (a), LST map (b).

The normalized multi-band drought index (NMDI) analysis indicates that the study area is under high moisture stress and severe land degradation. Accordingly, about 32.06%, 31.07%, and 22.15% fall into high, moderate, and low degradation levels, respectively (Table 13). The highly degraded land in the area indicates reduced soil moisture and subsequently declining vegetation cover. Notably, 9.6% of the area is very highly degraded, showing severe soil moisture loss and dryness, as well as substantial vegetation stress. Meanwhile, only 5.12% of the area is estimated to have a very low land degradation vulnerability level, covering limited localities and remains fragmented. The spatial distribution map clearly indicates that the northern, central, and northeastern parts of the study area are affected by high to very high land degradation (Fig 5a). The results generally indicate that more than 70% of the study area is under moderate to very high soil moisture stress. Soil moisture deficit and land surface dryness further facilitate the degradation rate. Hence, sustainable land and water management strategies should be implemented in the region to address the severity of moisture loss and the consequent land degradation.

**Table 13:** Normalized multi-band drought index (NMDI).

Classes	Score	Land degradation vulnerability level	Area (km^2^)	Area (%)
<0.5	1	Very low	135.05	5.12
0.5-0.55	2	Low	583.71	22.15
0.56-0.6	3	Moderate	818.87	31.07
0.61-0.7	4	High	844.82	32.06
>0.7	5	Very high	253	9.6
Total			2635.45	100

### 3.4 Land surface temperature (LST)

The spatial distribution of the land surface temperature analysis indicates that the study area is experiencing high thermal stress, mainly due to subsurface uplifting activities. Increased thermal stress leads to vegetation stress, loss of soil moisture, and weakening of the internal tension between soil grains. Ultimately, this makes the land surface easily susceptible to soil erosion and land degradation. Table 14 shows LST classes and their corresponding land degradation vulnerability level.

**Table 14:** Land surface temperature (LST).

Classes	Score	Land degradation vulnerability level	Area (km^2^)	Area (%)
<30	1	Very low	17.75	0.67
30-35	2	Low	266.43	10.11
35.1-40	3	Moderate	925.48	35.12
40.1-45	4	High	823.5	31.25
>45	5	Very high	602.29	22.85
Total			2635.45	100

The result showed that 35.12% of the land is moderately degraded, 31.25% is highly degraded, and 22.85% is under very high degradation. A high degradation level is observed in areas with low vegetation cover and increased soil moisture stress, which are attributed to elevated land surface temperatures. Bare lands and croplands are showing high to very high levels of thermal stress and degradation. The effect of LST is high in the northern, central, southern, and southwestern parts of the study region (Fig 5b).

In contrast, 10.11% and 0.67% of the study area are low- and very low-degraded, respectively. These areas are relatively good in their soil moisture availability and vegetation cover. In conclusion, the land surface temperature analysis indicates that more than 89% of the study area is moderately to very severely degraded attributed to thermal stress limiting the areas for vegetation cover and agricultural production. Nonetheless, promoting thermophilic (heat- and temperature-resistant) grasses and crops could be best management strategies for the area.

### 3.5 Soil salinity

Soil salinity is a chemical indicator of land degradation, commonly observed in arid and semi-arid areas where evaporation exceeds rainfall. Soil salinity also occurs in areas with poor drainage, improper irrigation systems, and saline groundwater. It is identified in the field by observing indicators of saline environments, such as the presence of halophytic (salt-tolerant) plants, the exposure of white salt crusts at the topsoil layer (Fig 7), reduced vegetation coverage, and poor crop and vegetation growth. The presence of salinity or sodicity in a given area is a triggering factor for soil dispersion and loss of soil structural integrity. A chemical imbalance decreases the resistance of soil grains to water shear force, ultimately leading to soil erosion and severe land degradation.

**Fig 6:**
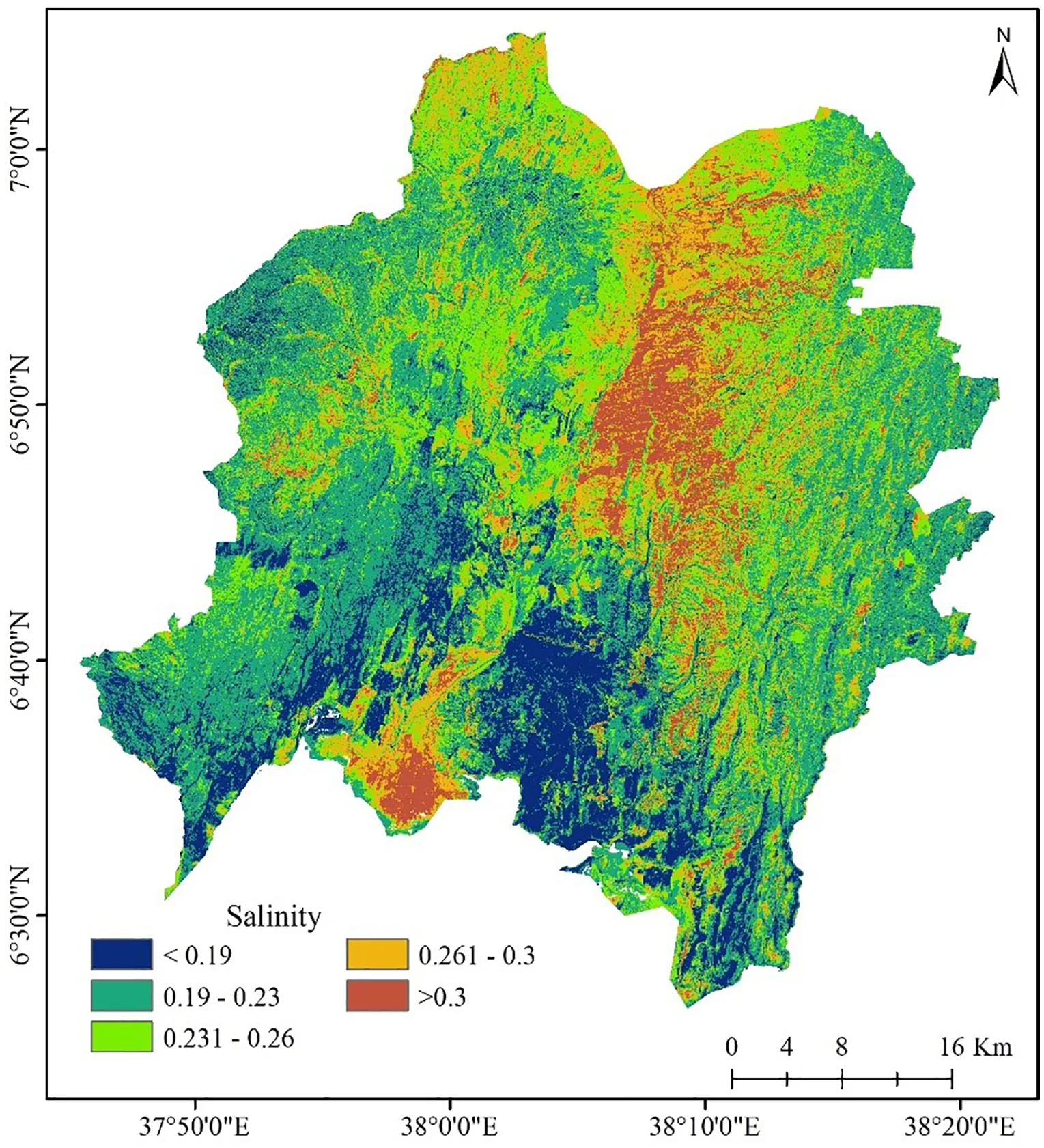
Soil salinity map of the study area.

The soil salinity analysis of the study area shows significant salt deposits, which facilitate land degradation vulnerability. About 46.36% of the study area is moderately to very saline and degraded (Table 15). Of this, 570.06 km2 (21.63%) of the study area is in the high to very high salinity range, forming gully-erosion and rift-fragile morphologies. Highly saline areas were observed in the northern, central, and southern parts of the study area (Fig 6).

**Table 15:** Soil salinity intensity.

Classes	Score	Land degradation vulnerability level	Area (km^2^)	Area (%)
<0.19	1	Very low	382.52	14.51
0.19-0.23	2	Low	1031.22	39.13
0.231-0.26	3	Moderate	651.65	24.73
0.261-0.3	4	High	373.23	14.16
>0.3	5	Very high	196.83	7.47
Total			2635.45	100

**Fig 7:**
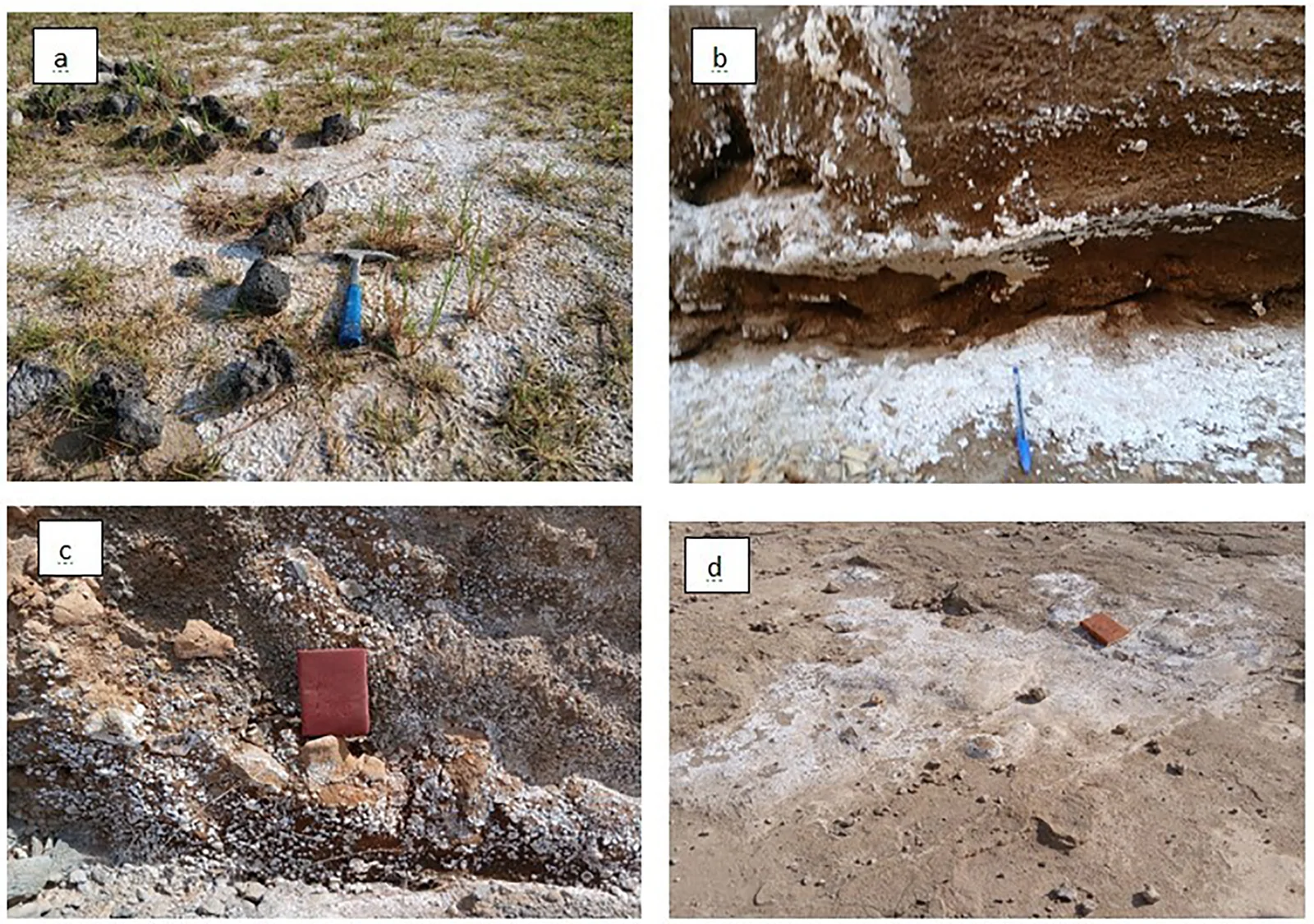
Indicators of severe land degradation through soil salinization in the study area: (a) extensive white salt crust formation on sparsely grass; (b) subsurface salt accumulation and sub-surface efflorescence within an exposed soil profile; (c) dense, crystalline salt precipitates along a soil/rock matrix; and (d) prominent surface crusting, sealing, and salt efflorescence on barren topsoil.

These areas are in a critical state of land degradation, requiring immediate actions such as promoting physical and biological reclamation to support safe and sustainable land management practices in the rift region and address the expansion of rills and gullies. On the other hand, 39.13% of the area is under low land degradation vulnerability levels, ranging from a salinity class of 0.19 to 0.23. The remaining 14.51% of the area is less saline and very low saline. Overall, the concentration of saline hotspots in the area indicates that the observed chemical degradation is resulted in soil structure disturbance. Therefore, immediate soil management and conservation strategies are necessary to minimize soil erodibility and land degradation (Fig 7).

### 3.6 Soil erosion

a. **R-Factor**

**Fig 8:**
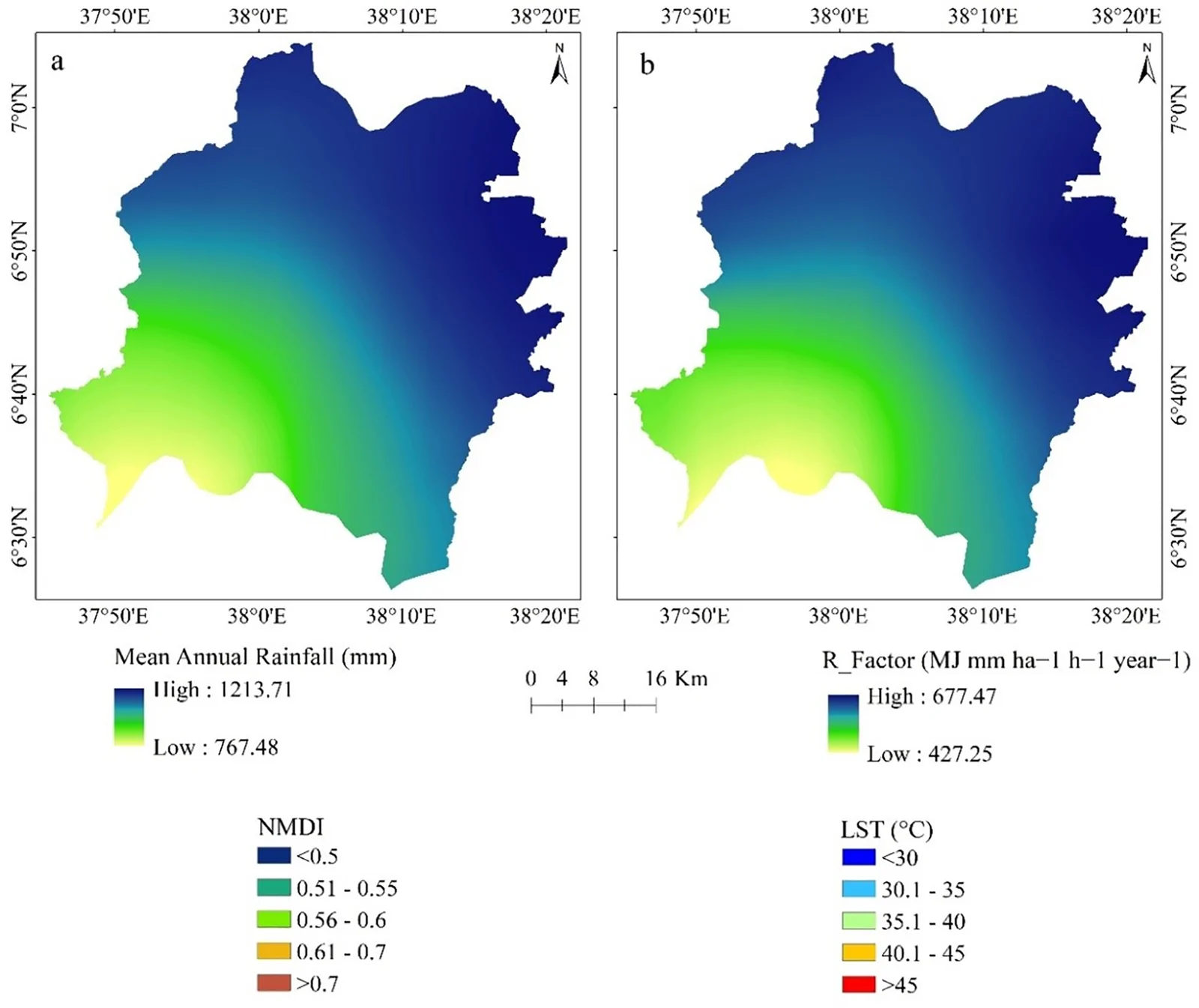
Mean Annual Rainfall map (a), R factor map (b).

The rainfall erosivity (R) factor map shows the spatial variation in rainfall intensity and its erosivity within the study area. The mean annual rainfall distribution in the study area ranges from 767.48 mm to 1213.71 mm (Fig 8a). The R-factor values range from 427.25 MJ mm ha^-1^ h^-1^ yr^-1^ to 677.47 MJ mm ha^-1^ h^-1^ yr^-1^ (Fig 8b). The rainfall map and the R-factor map show spatial resemblance, indicating a positive relationship between annual precipitation and rainfall erosivity. Mostly, areas receiving high rainfall intensity are susceptible to soil detachment and sediment transport, resulting in increased land degradation vulnerability over time.

The northern, central, north-eastern, and eastern parts of the study area exhibit relatively high R-factor values, indicating rainfall-induced soil erosion. These areas could be affected by sheet, rill, and gully erosion due to high-intensity rainfall-induced runoff. Interactions between rainfall and weathered, tectonically disturbed geological materials exacerbate erosion. In addition, the observed salinity, sodicity, and hydrothermal alteration reduce the overall stability of the soil materials by creating surface sealing and runoff, making the area highly vulnerable to erosion. Elevated land surface temperatures also cause soil erosion by promoting dryness and moisture stress. Moisture stress in the area and agricultural land expansion increase soil erosion, resulting in a high degree of sediment transport. In contrast, the southern and southwestern parts of the study area experienced lower erosivity values due to lower rainfall-induced erosion potential. Soil and water conservation practices, watershed management, afforestation, salinity and sodicity treatment, and other integrated management strategies are required to minimize soil erosion and the risk of future land degradation.

b. **K-Factor**

**Fig 9:**
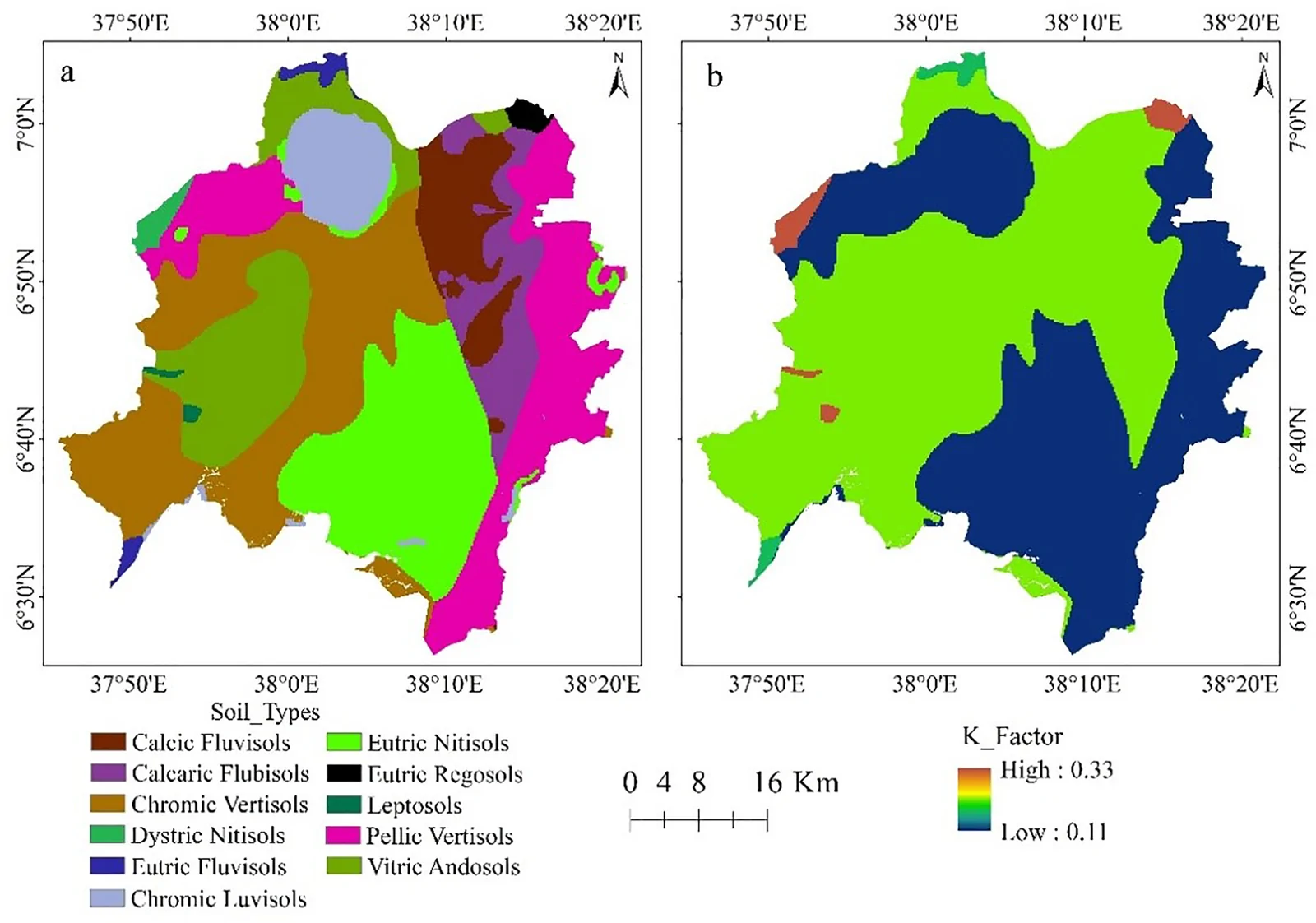
Soil types map (a), K-factor map (b).

The spatial distribution of erodibility of various soil groups is illustrated in the K-factor map of the study area (Fig 9). It clearly states the susceptibility of soil particles to detach and be transported by intensive rainfall and surface runoff. The findings of this study show that the soil erodibility (K-factor) values of the study area fall within the range of 0.11 ha h (ha MJ mm)^-1^ to 0.33 ha h (ha MJ mm)^-1^ (Fig 9b). This refers to the soils in the study area having varying degrees of erosion sensitivity. High K-factor values indicate that the areas are more vulnerable to soil erosion due to tectonic disturbance of soil materials, lower organic content availability, and low permeability.

In contrast, areas with lower K-factor values are comparatively erosion-resistant due to their high clay and organic matter content, stable soil structure, and greater infiltration capacity. In contrast, the soils in the northern and western parts of the study area are highly erodible, whereas those in the eastern, southeastern, and northwestern portions are low erodible. The salinity, sodicity, and anthropogenic factors, such as inappropriate cultivation, overgrazing, and improper land management, observed in the study area disturb soil stability and ultimately increase soil erodibility. Thus, the K-factor result suggests that soil properties are determinants of erosion processes and land degradation. It also highlights the importance of soil-specific conservation measures and land management strategies to mitigate the risk of soil erosion in the area. Areas with a high degree of erodibility require immediate erosion control measures, such as afforestation, terracing, and other soil-stabilisation measures, to reduce degradation and sustain the ecosystem.

c. **LS-Factor**

**Fig 10:**
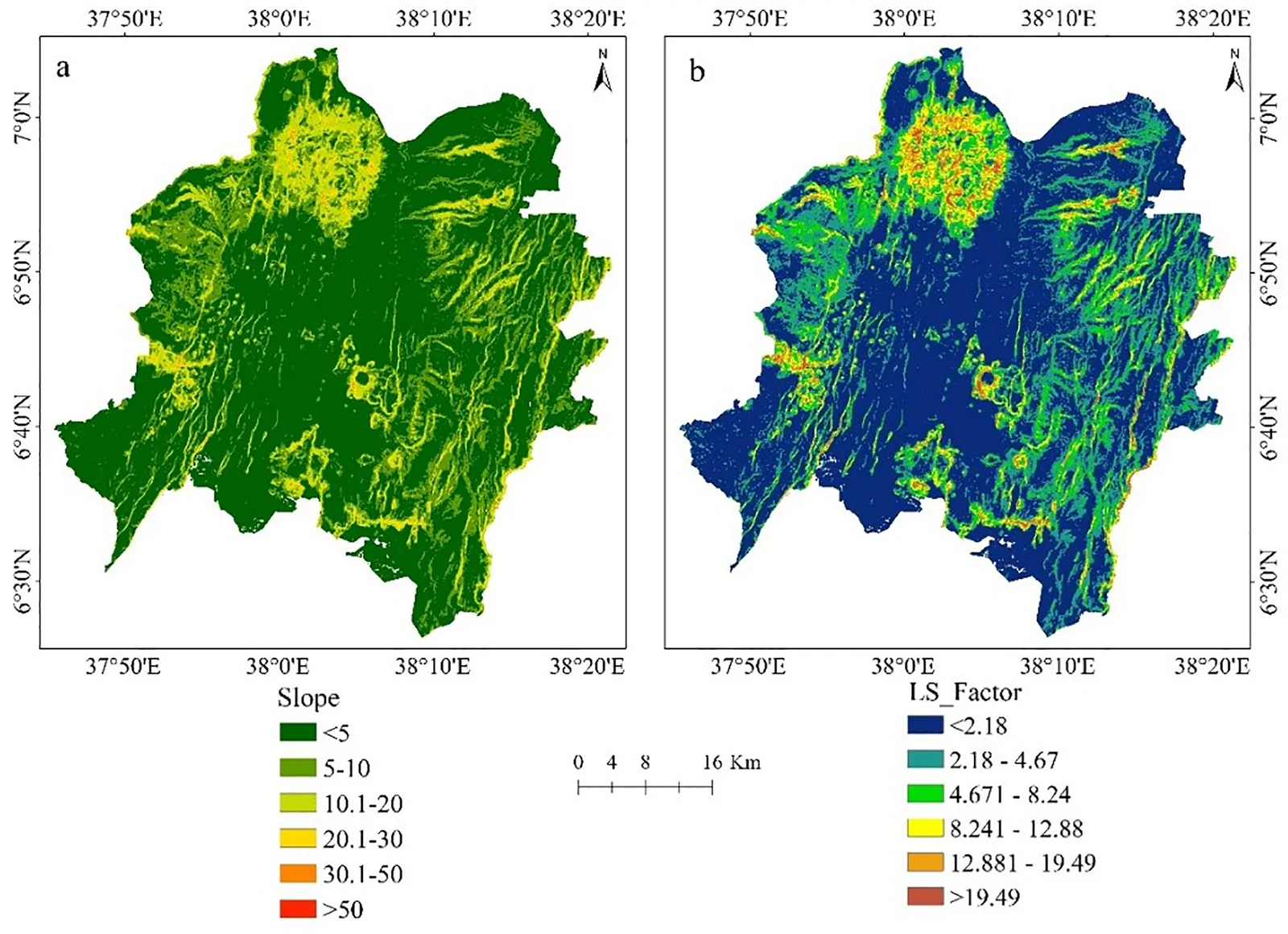
Slope map (a), LS factor map (b).

The slope length steepness factor (LS) is an important parameter for modeling soil erosion risk. The slope length steepness (LS) factor map of the study area shows the topographical influence on soil erosion. In this study, slope gradient and slope length are considered to assess spatial variations in soil erosion. These factors govern the area’s soil erosion susceptibility by influencing surface runoff during heavy rainfall. In general, steeper and longer slopes (high LS) are highly susceptible to erosion. Gentle, short slopes or slopes with lower LS values are less vulnerable to runoff generation and surface soil erosion. The slope map shows that the dominant portions of the study area have gentle to moderate slopes (<100), whereas the remaining areas are steep to very steep, consisting of >2^00^ (Fig 10a). The LS-factor values of the area fall within the range of less than 2.18 to greater than 19.49 (Fig 10b). This means that erosion risks due to the LS factor in the study area are highly spatially variable. High LS values are shown in the northern, northeastern, western, and central parts of the study area. Finally, the LS-factor result indicates that soil conservation measures based on rifts and slopes, along with watershed management, are required to reduce erosion risk in the area.

d. **C-Factor**

**Fig 11:**
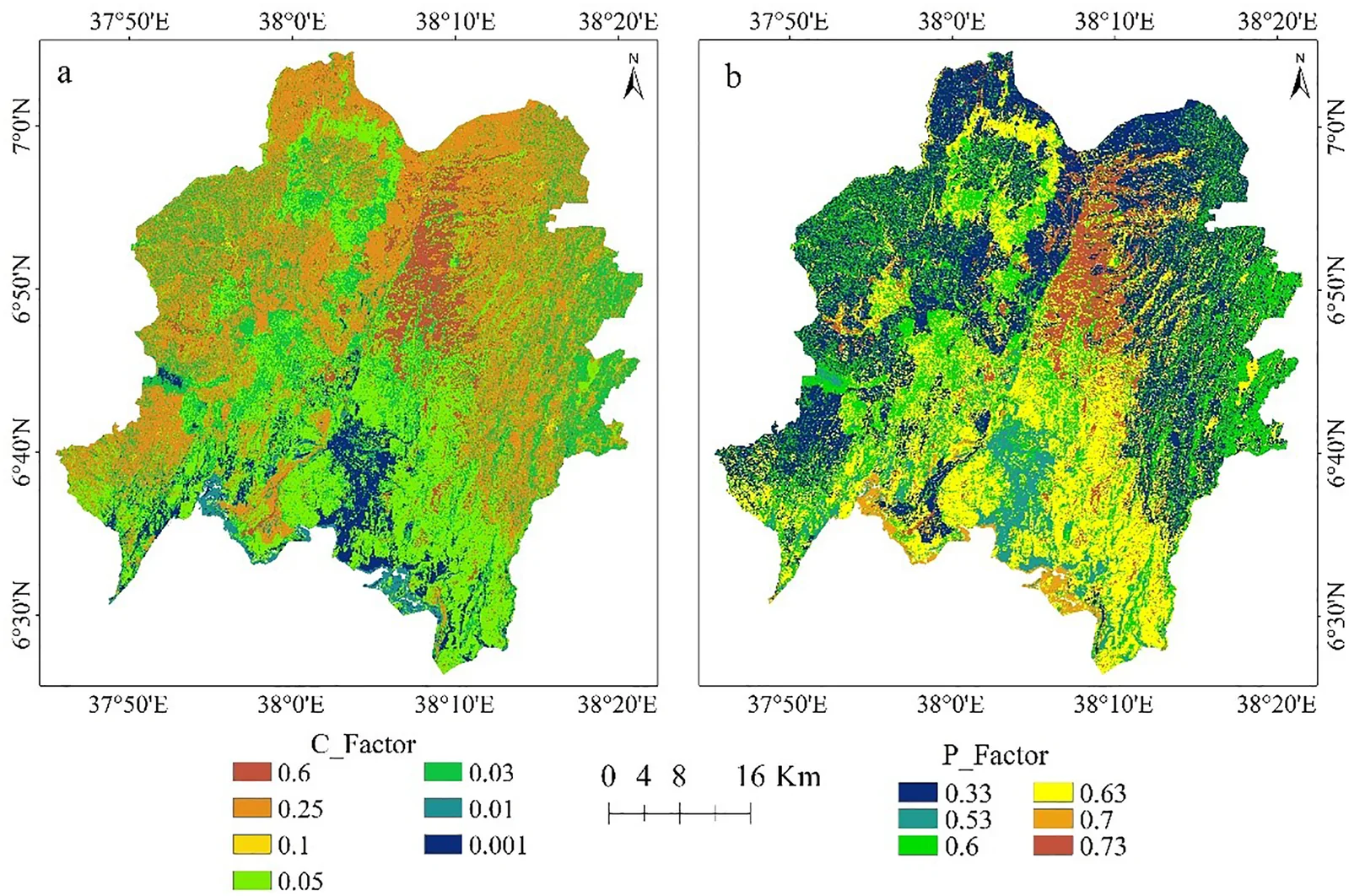
C-factor map (a), P-factor map (b).

The cover management factor (C-factor) is an important parameter for estimating and managing soil erosion. The cover management factor map illustrates the roles of vegetation cover, land use, and land management practices in estimating soil erosion. It shows the effectiveness of vegetation density and surface cover in reducing rainfall and runoff impacts on soil particle detachment and transport. Different crop residues also have the potential to reduce soil erosion by decreasing raindrop impacts, increasing infiltration rate, and increasing soil moisture. Cover management factor (C-factor) values for this study range from near 0 for protected areas to 1 for bare, highly exposed areas. The C-factor values range from 0.001 to 0.6, indicating low to moderate values (Fig 11a). Densely vegetated areas, grasslands, and forestlands have lower C-factor values and minimal erosion risk.

On the other hand, sparsely vegetated areas, cultivated lands, tectonically disturbed landscapes, and degraded and bare lands exhibit higher C-factor values, indicating a high potential for soil erosion and land degradation. The erosion vulnerability level of agricultural lands is relatively lower than that of bare lands because of the presence of crop cover, which reduces rainfall erosivity and soil erodibility. Agricultural lands have better crop cover, which can help decrease surface soil erosion. Commonly, water bodies have a C-factor value of 0 and are not considered in soil erosion models. Thermally stressed and soil moisture-depleted areas have high C-factor values. Likewise, soil salinity and hydrothermal stress also increase the C-factor values of the area. In addition, croplands and settlement expansion, deforestation, and overgrazing aggravate soil erodibility by exposing areas to erosive agents, resulting in high C-factor values. Therefore, deep insight into the spatial distribution of C-factor values helps adopt site-specific measurement mechanisms, such as afforestation, reforestation, and sustainable land-use management strategies, to reduce the area’s erosion and land-degradation vulnerability.

e. **P-Factor**

RUSLE modelling uses the support practice factor to quantify the influence of multiple conservation practices. These conservation practices have the potential to reduce the effects of erosive forces and sediment transport in a given area. Bio-fencing, strip cropping, terracing, contour farming, bund construction, and other engineering measures are some of the effective mechanisms for soil conservation through reducing runoff velocity, improving infiltration and soil moisture, and rehabilitating degraded lands. The support practice factor (P) map shows how soil and water conservation measures are effective in reducing erosion and degradation. The P-factor values in the study range from 0.33 to 0.73 (Fig 11b). This value refers to the spatial variation in the effectiveness of conservation measures across the area. The lower the P-factor values, the more effective land management and conservation practices are at minimizing soil erosion in the area. On the contrary, the higher the P-factor values, the more limited and ineffective the land management and conservation measures are, exacerbating soil erosion risk and land degradation. Cultivated and degraded lands with inadequate soil conservation measures exhibit high P-factor values and cover the northeastern, central, and southern portions of the study area. In the study area, the dynamic nature of the rift and anthropogenic factors rendered soil conservation efforts weak and ineffective. As with man-made activities, tectonic disturbances, salinity, and hydrothermal alterations contribute to soil erosion and land degradation. Hence, the findings of this study indicate the need for integrated supporting conservation measures in highly soil-erosion and land-degradation-vulnerable areas to overcome the observed risk. Implementing effective long-term land management strategies helps protect the area from erosion.

f. **Annual Soil Erosion Estimation**

**Fig 12:**
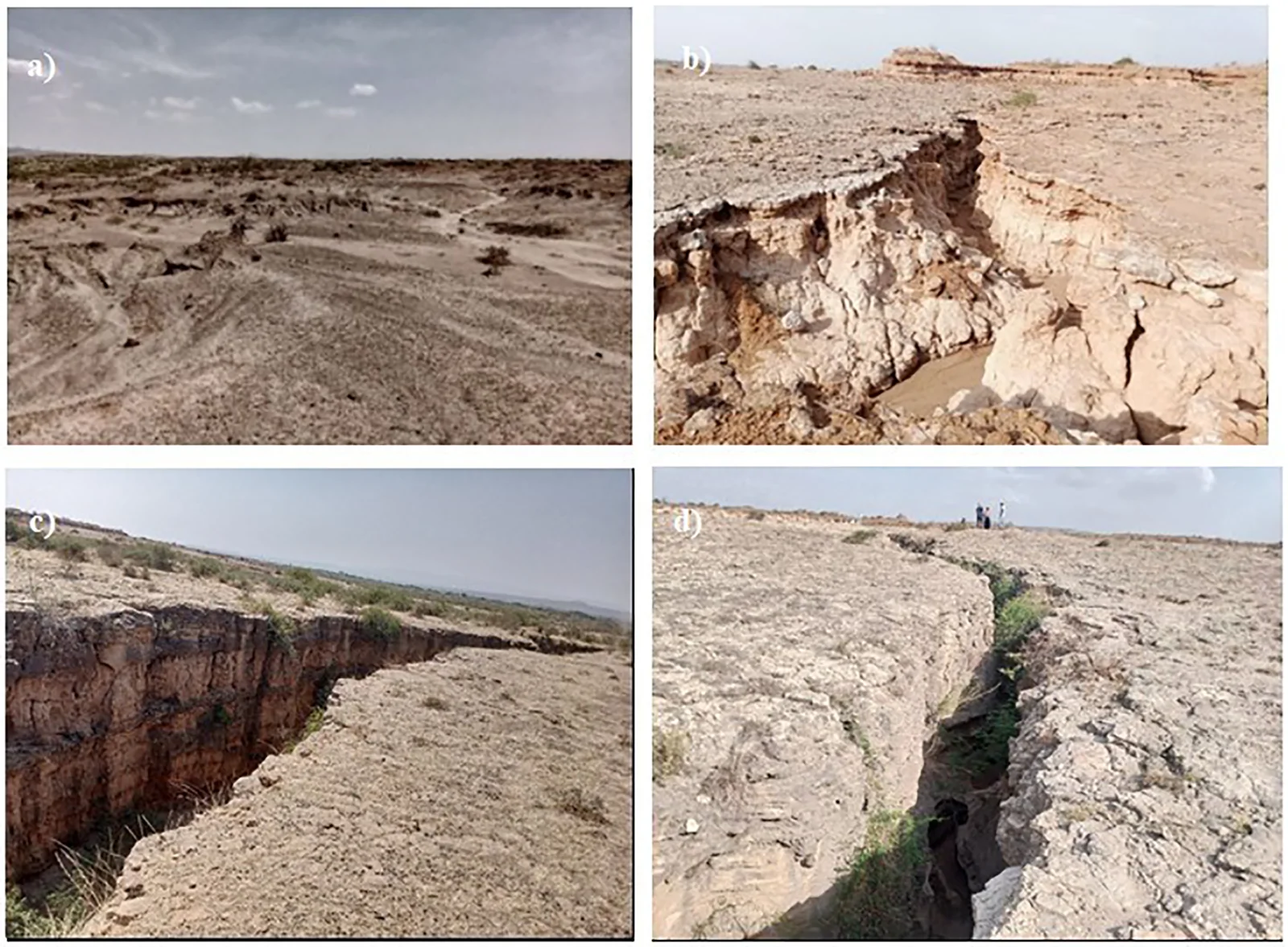
Different types and stages of soil erosion observed within the study area: (a) widespread sheet erosion leading to extensive topsoil loss and a severely degraded landscape; (b) transition from advanced rill erosion to early gully formation with severe bank destabilization; and (c, d) deeply incised, active gully networks exhibiting significant vertical wall exposure and headward propagation.

**Fig 13:**
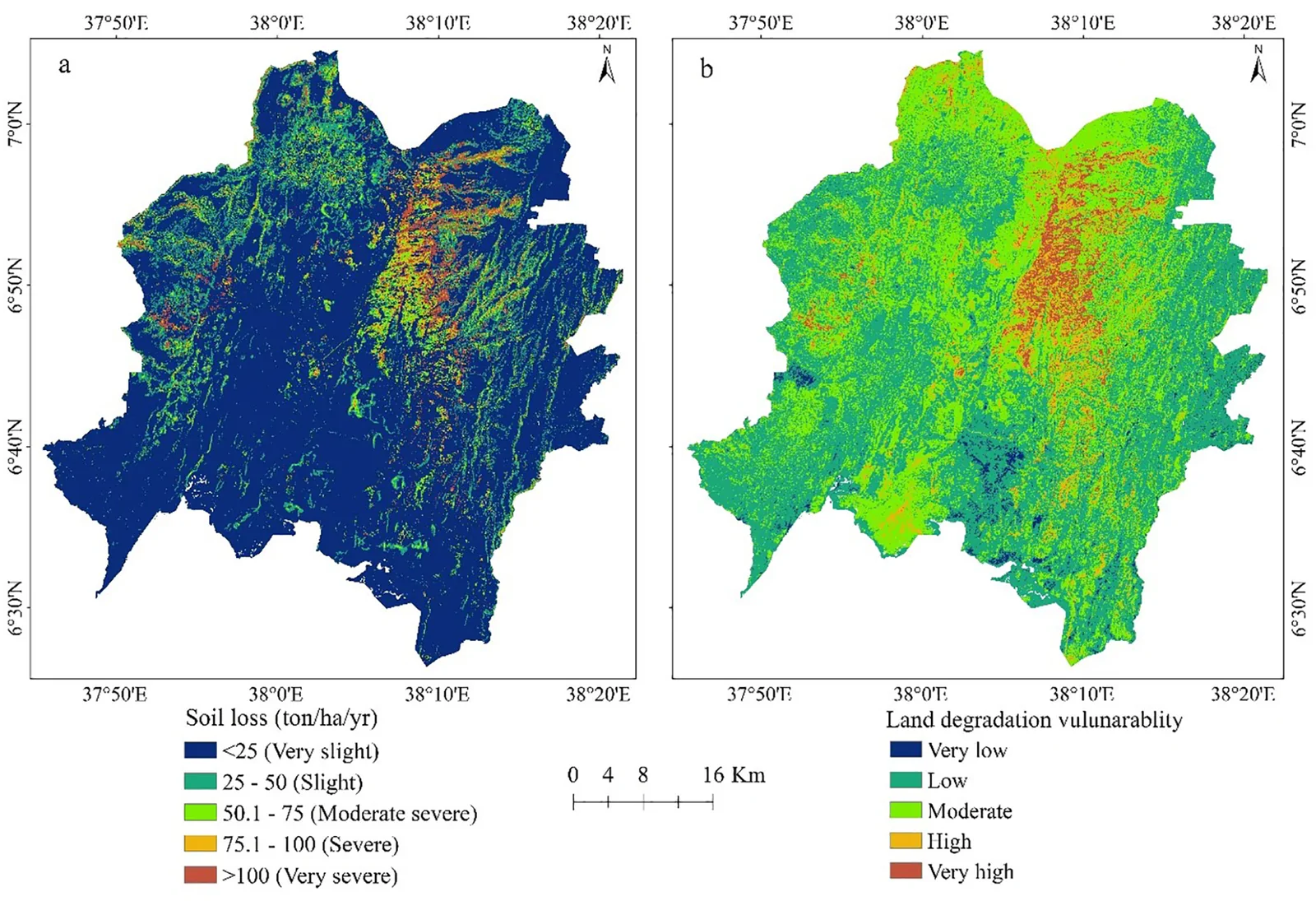
Soil loss estimation map (a), Land degradation vulnerability map (b).

The annual soil loss in the study area was analyzed using the empirical RUSLE model. The RUSLE-based soil erosion analysis indicates that about 3.6% (95.05 km^2^), 2.35% (95.99 km^2^), and 4.74% (124.88 km^2^) are very severely, severely, and moderately eroded, respectively (Table 16). These areas lie in active tectonic and fault-controlled environments with high deposits of loose volcanic ash and weathered geomaterials. They are highly disturbed, salinized, less vegetated, and easily exposed to high thermal stress and soil moisture depletion, which eventually leads to soil erosion due to weakened soil structure. Sheet erosion and high-depth rill and gully erosion were observed in cultivated lands in the study area (Fig 12). These areas require immediate intervention to address the problem. The result further shows that severe- and very-severe erosion zones are concentrated largely in the central and northeastern parts of the study area (Fig 13a). On the other hand, 12.22% (321.98 km2) and 77.09% (2031.55 km2) fall into the slightly eroded and very slightly eroded categories, with annual soil loss of 25–50 t ha-1 yr-1 and <25 t ha-1 yr-1, respectively, indicating that land degradation is low in these areas. Although the larger areas show very slight erosion, the observed severely eroded areas are strong indicators of ongoing soil erosion and land degradation in the study area. The presence of tectonic disturbance, land-use change, and the absence of adequate rift-based soil conservation and mitigation measures are the main causes of the observed high degree of soil loss. It is impossible to minimize soil erosion and its rate of degradation unless effective intervention measures are implemented across the entire study area. Hence, integrated soil and water conservation measures, afforestation, salinity control, safe irrigation practices, and sustainable climate-adaptive land management strategies are required to curtail future soil loss and land degradation in the region.

**Table 16:** Soil loss estimation (ton ha^-1^ yr^-1^).

Classes	Score	Land degradation vulnerability level	Area (km^2^)	Area (%)
<25	1	Very slight	2031.55	77.09
25-50	2	Slight	321.98	12.22
50.1-75	3	Moderate severe	124.88	4.74
75.1-100	4	Severe	61.99	2.35
>100	5	Very severe	95.05	3.6
Total			2635.45	100

### 3.7 Land degradation vulnerability mapping

The land degradation vulnerability map and corresponding spatial statistics reveal that 10.03% (264.39 km^2^) of the study area falls under high (6.31%) and very high (3.72%) land degradation vulnerability levels. In comparison, 38.14% (1005.12 km^2^) exhibits moderate vulnerability (Table 17). The spatial distribution indicates that the structurally controlled central and northeastern parts of the area are particularly exposed to high and very high degradation risks (Fig 13b). It is worth noting that while water-driven physical soil erosion (RUSLE) is topographically restricted to steeper slopes, thereby affecting a smaller areal extent (10.69%), the overall composite LDV model integrates chemical (salinity), biological (vegetation condition), and thermal (LST, NMDI) stress factors alongside RUSLE. Consequently, expansive flat lowlands that exhibit minimal physical water erosion are still estimated to have moderate-to-high overall degradation vulnerability due to severe soil salinization, sodicity, and hydrothermal stress. From a geological perspective, the very high degradation zones reflect the combined impacts of shallow groundwater salinization, endogenic hydrothermal alteration, and elevated land-surface temperatures, which are characteristic of active rift environments. Upward capillary transport of shallow saline groundwater, driven by high evaporation rates, promotes surface salt accumulation. Concurrently, ascending hydrothermal solutions accelerate the weathering of subsurface soil and rock. In areas with sparse vegetation cover and exposed bare land, elevated land surface temperatures intensify evaporation, depleting soil moisture. The central rift area exhibiting high degradation vulnerability is characterized by elevated thermal stress and severe localized soil loss. Similarly, the southern sector near Lake Abaya shows moderate-to-high degradation, with pronounced thermal anomalies compared to densely vegetated zones. Weakly consolidated volcanic and pyroclastic deposits across the region are highly weathered and structurally susceptible to degradation. Additionally, physical crusting induced by salinity, sodicity, and rapid drying severely restricts infiltration capacity, escalates surface runoff, exacerbates topsoil stripping and rill formation, and ultimately degrades long-term soil productivity.

**Table 17:** Land degradation vulnerability in the study area.

Land degradation vulnerability level	Score	Area (km^2^)	Area (%)
Very low	1	44.31	1.68
Low	2	1321.63	50.15
Moderate	3	1005.12	38.14
High	4	166.25	6.31
Very high	5	98.14	3.72
Total		2635.45	100

**Fig 14:**
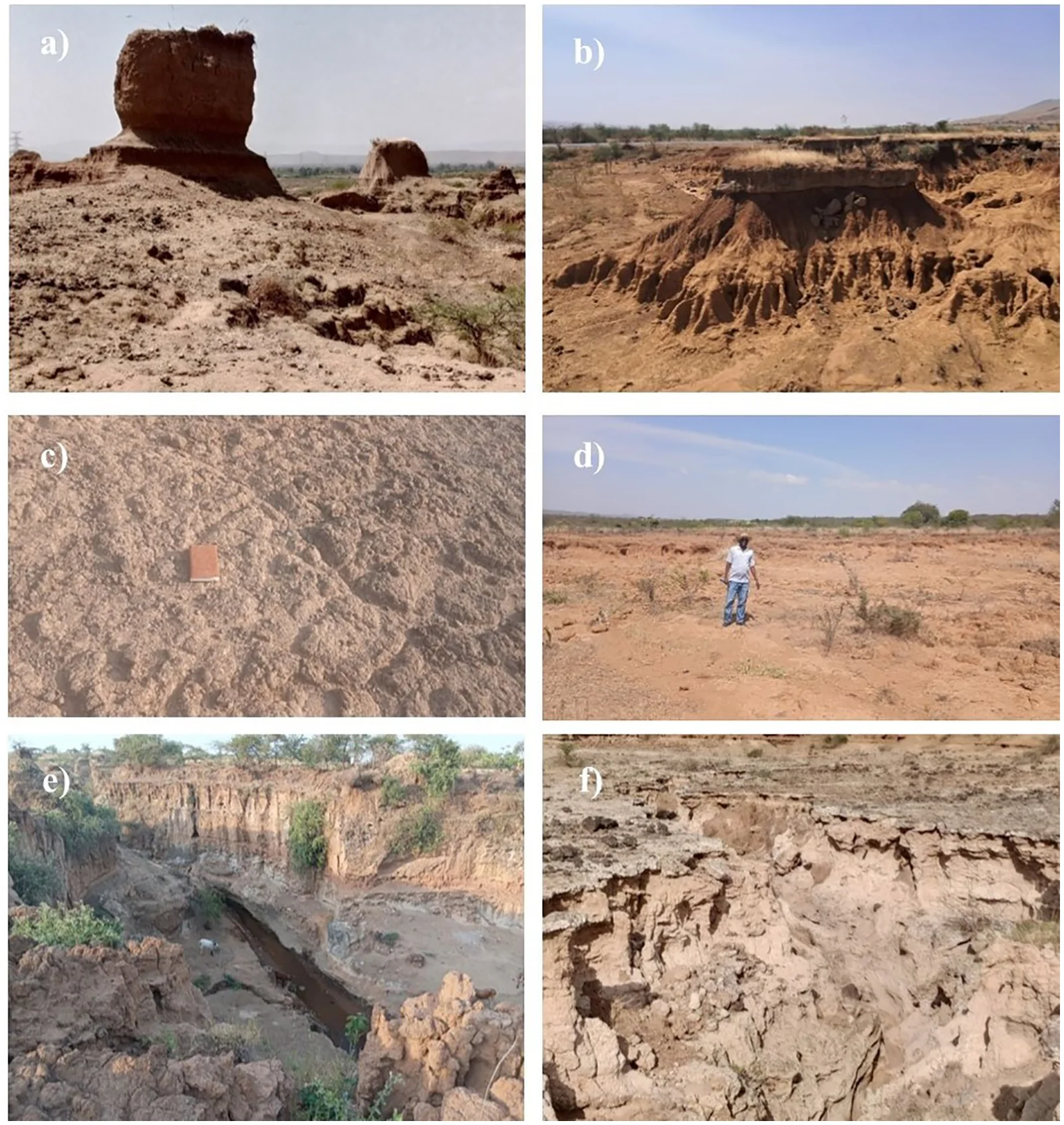
Evidence of severe land degradation and soil erosion features within the study area: (a) pedestals indicating significant sheet and rill erosion; (b) advanced rill and miniature gully formation on exposed slopes; (c) extensive surface crusting and sealing; (d) barren, degraded land showing a loss of vegetative cover; (e) deep, active gully system causing severe terrain dissection; and (f) severe bank collapse and headward erosion at a gully head.

Of the total area, 50.15% (1321.63 km2) is low-degraded, and 1.68% (44.31 km2) is very low-degraded (Table 17). These areas are comparatively vegetated, minimally disturbed, and less eroded. The overall result reveals that moderate-to-high and very high degraded areas will be severely degraded if proper soil conservation and land management strategies are not designed and implemented (Fig 14). Therefore, the current and future degradation risk can be minimized by integrating environmental restoration and implementing land management strategies across the area.

### 3.8 Validations and accuracy assessment

**Fig 15:**
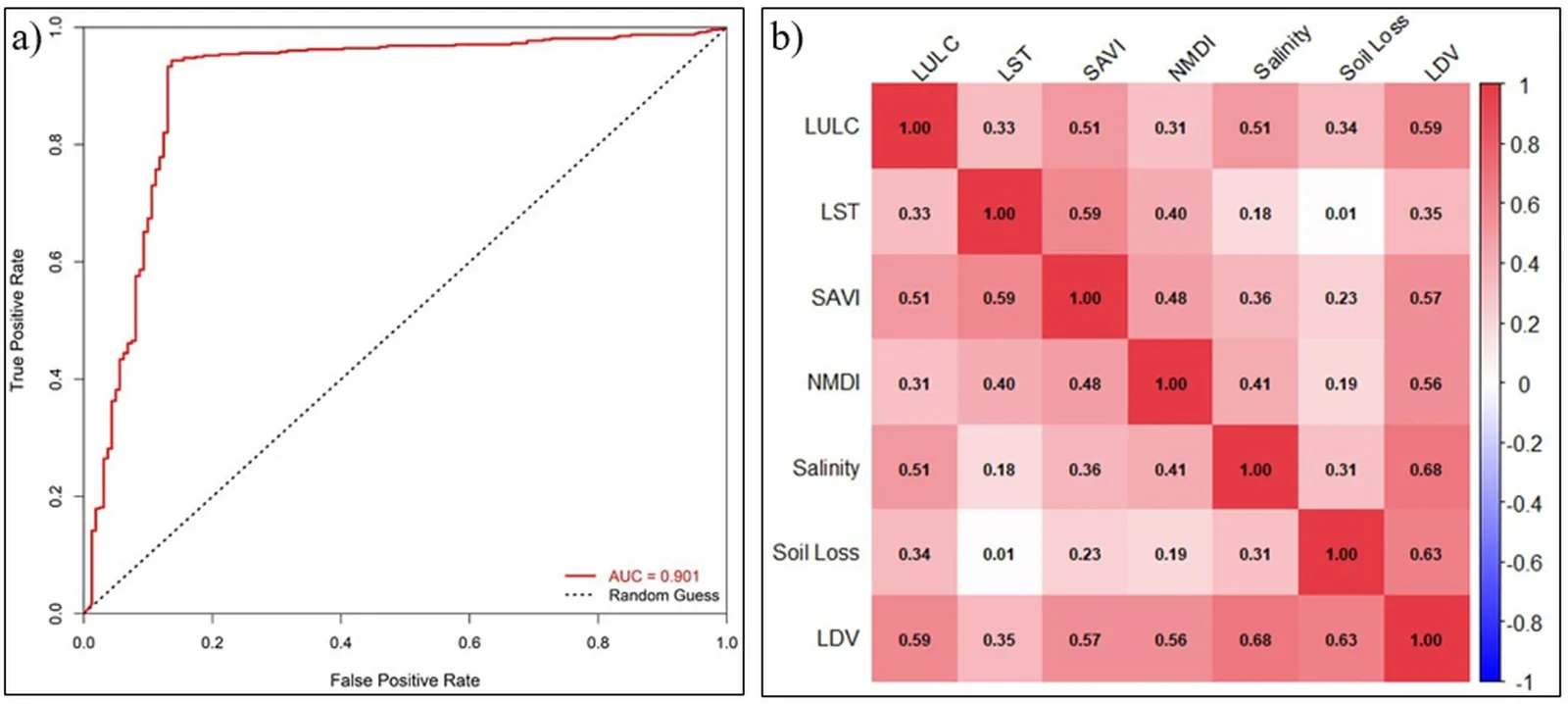
a) ROC and AUC Curve for Validation, b) Spearman’s rank correlation heatmap between individual factors and the final land degradation vulnerability map.

The LDV validation results indicate an overall accuracy of 92.34% and a kappa coefficient of 0.87. In terms of precision, the model performs very well, with 95.4% of the area identified as vulnerable on the LDV map. In contrast, the recall metric shows that 94.2% of the area identified as vulnerable on the ground is captured. The F1-score is 0.948, reflecting a good balance between precision and recall. Additionally, the ROC-AUC value of 0.901 demonstrates the model’s strong capability to discriminate between vulnerable and non-vulnerable areas (Fig 15). Overall, these validation results confirm the model’s accuracy and reliability for the LDV map.

### 3.9 Spearman’s rank correlation heatmap

The Spearman’s correlation coefficient matrix indicated a positive relationship between land degradation indicators and LDV, with ρ values ranging from 0.35 to 0.68 (Fig 15). According to Spearman’s correlation coefficient, salinity exhibited a moderate association (ρ = 0.68) with LDV, followed by LULC (ρ = 0.59), SAVI (ρ = 0.57), and NMDI (ρ = 0.56). On the other hand, soil loss and LST showed lower correlations with LDV (ρ = 0.43 and 0.35, respectively). SAVI shows a moderate association with LST (ρ = 0.59) and LULC (ρ = 0.51), while LST shows a weak correlation with soil loss (ρ = 0.01 and salinity (ρ = 0.18). The results showed that salinity, soil erosion, LULC, SAVI, and NMDI are strong indicators of land degradation, while LST is a significant indicator but has slightly lower correlations. These indicators are crucial for assessing land degradation vulnerability in the study area.

## 4 Discussion

Land degradation is a common environmental challenge that affects socio-economic development and environmental sustainability [6,80]. Integrating multi-source Earth observation data, machine learning algorithms, and RUSLE models was used to map land degradation vulnerability in the Southern Main Ethiopian Rift. The study results highlight that the areas are vulnerable to land degradation driven by natural and anthropogenic factors, with the extent and severity varying from place to place. The results further indicated that the degradation problem in the study area is the combined impact of salinity, sodicity, hydrothermal stress, high land surface temperature, soil erosion, vegetation stress, and tectonic and human-induced disturbances. The interactions among these several factors disclose the sensitivity of active rift regions to both natural and man-made disturbances.

The land use/land cover analysis shows that soil erosion and land degradation problems in the study area are higher in bare lands and cultivated lands, where soil moisture and vegetation cover are lower than in forests, wetlands, and shrub lands. Comparable findings have been noted in other regions of Ethiopia, where the expansion of agricultural land and the decrease in vegetation cover have significantly increased soil erosion and land degradation [11,81,82]. The SAVI and NMDI analyses also show that large portions of the study area exhibit moderate to very high land degradation vulnerability. This result refers to soil moisture shortage and reduced vegetation cover, which facilitate reduced soil cohesion, loss of soil aggregate stability, and increased sediment transport by rainfall and runoff [83,84]. These results demonstrate that the complex interactions among soil erosion, topography, vegetation condition, and moisture availability in the Southern Main Ethiopian Rift cannot explain the spatial pattern of land degradation vulnerability.

The main causes of vegetation cover loss in the study area are salinity, high thermal stress, tectonic disturbance, and moisture deficit. In turn, elevated thermal stress maximizes evaporation rates and reduces soil moisture availability. Similar studies have been reported in arid and semi-arid regions worldwide, where elevated temperatures accelerate the loss of soil moisture and the depletion of water storage for plant life, exacerbating vegetation loss and enhancing bare land [85,86]. Owing to the above factors, areas with insufficient vegetation cover are degrading and losing environmental resilience, ultimately leading to reduced agricultural productivity and exacerbating food insecurity due to soil fertility loss. The land surface temperature analysis is primarily conducted to illustrate the role of thermal stress in accelerating soil erosion and land degradation by intensifying evaporation rates, drying soils, and destabilizing soil structure. Elevated land surface temperatures are observed in sparsely vegetated, moisture-deficient, and salinized areas. Hydrothermal activity in active rifts is also a major driver of rising surface temperatures and mineral alteration, affecting crop growth. The alteration again weakens soil cohesion and weathered geological materials deposited in the area, eventually increasing erosion and degradation. The altered pumice and travertine formations in the study area are the best indicators of interactions between hot fluids and geological materials that cause environmental degradation. Taken together, the LULC, SAVI, NMDI, and LST results indicate that vegetation degradation, moisture stress, and thermal stress operate as interacting rather than isolated processes in the study area. This interaction implies that land-management interventions should simultaneously improve vegetation cover and soil-moisture retention while accounting for locally elevated thermal and hydrothermal stress, particularly in cultivated and sparsely vegetated areas.

Along with elevated land surface temperature, salinity is one of the major causes of soil erosion and land degradation vulnerability in the study area. Salinity problems are commonly observed in hydrothermally stressed, low-lying graben environments where high precipitation is induced by substantial evaporation. The vegetation coverage is too low in these zones. The salinity analysis shows that salt crusts and soil surface sealing in the study area reduce infiltration rates, increase vegetation stress, and ultimately affect the sustainability of agricultural productivity [87]. Subsequently, instability, dispersion, and transport of soil particles occur due to runoff generated during rainfall events. Similar findings have been reported in previous studies, which identified salinity, sodicity, and limited vegetation cover as driving factors of runoff generation and soil water erosion [34,79,88]. Therefore, deep erosional features, such as rills and gullies, are widespread across the study area. This finding indicates that erosion-control measures alone may be insufficient in the most affected zones unless they are accompanied by salinity- and sodicity-management measures, physical and biological reclamation, and interventions that restore soil structure and infiltration capacity. In relation to the central research objective, this result demonstrates that salinity is not merely a secondary soil-quality issue but a major component of land-degradation vulnerability in the study area, linking the hydrothermal and geochemical characteristics of the active rift environment with physical erosion and vegetation degradation.

The RUSLE-based soil erosion assessment used various factors, such as rainfall erosivity, soil erodibility, slope length and steepness, cover management, and support practice, to predict and map erosion vulnerability in the study area. Many studies conducted in Ethiopia and other countries have employed these five factors to assess soil erosion risk, quantify soil loss, and delineate erosion-prone areas [58,89,90]. The findings of these studies show that rainfall erosivity, slope length and steepness, soil properties, land cover conditions, and support practices are determinants of soil erosion susceptibility. The differences in erosion severity among the study areas may be due to variations in climate, topography, land-use practices, vegetation cover, and implemented conservation measures [58,91–93]. In this study, the applied RUSLE model clearly identifies localized areas with severe and very severe soil loss. The study area consists of highly weathered geological materials and poor vegetation cover relative to other localities. Besides, a large portion of the area is at a slight risk of erosion. The R-factor result confirms that areas receiving high rainfall are more vulnerable to runoff-driven soil erosion. Likewise, the K-factor values indicate that areas with loose, disturbed soils are susceptible to erosion. The erodibility potential depends on environmental conditions and the nature of the erosive agent. The structure and texture of soils, organic matter content, slope, rainfall magnitude and frequency, permeability, vegetation coverage, and land management practices are some of the determinants of soil erodibility. Similarly, the LS-factor analysis indicates that the topography of an area controls erosion and degradation rate. Generally, runoff and sediment transport are higher on steep slopes than on gentle slopes because of higher flow velocity and greater erosive power [94–97]. However, the gentle slopes in the study area are severely eroded by salinity, hydrothermal stress, and surface soil crusting. In the context of the study objective, the RUSLE result therefore quantifies the physical water-erosion component of land degradation, while also showing that RUSLE alone cannot account for the full degradation pattern observed in this salinity- and hydrothermally stressed landscape. This is because of reduced infiltration capacity and increased runoff caused by soil surface sealing and salt-induced crusting. The C-factor and P-factor analysis indicates that poor vegetation cover and limited conservation practices in the study area are also the main causes of the observed soil erosion and land degradation. Local communities adopted some conservation practices. However, the effectiveness of the conservation measures in controlling erosion is minimal due to ongoing geological processes. This shows that soil conservation measures should be well-integrated with the rift-specific land management strategies in the area.

The validation LDV model results indicate that salinity and vegetation- and moisture-related conditions are particularly important in explaining the spatial structure of composite land degradation vulnerability. At the same time, soil erosion and thermal stress remain relevant components of the integrated degradation process. The combined validation and correlation results therefore support the use of a multi-indicator framework rather than relying on a single degradation proxy to identify priority areas in this hydrothermally and salinity-stressed rift environment.

The final land degradation vulnerability result indicates that the central and northeastern parts of the study area are the most erosion- and degradation-vulnerable zones, requiring immediate intervention with soil conservation and land management strategies, in collaboration with the local community and concerned bodies. An important interpretation of this result is that the spatial extent of moderate-to-very high composite land degradation vulnerability is substantially greater than the area affected by moderate-to-very severe RUSLE-estimated water erosion alone. This difference occurs because the final LDV framework represents multiple interacting degradation pathways; consequently, relatively flat or weakly eroded areas can still be classified as vulnerable where salinity, sodicity, vegetation stress, moisture deficit, and elevated land surface temperature are pronounced. The result therefore demonstrates that physical soil-loss estimates alone do not fully represent degradation vulnerability in this active rift environment and supports the use of a multi-indicator assessment for prioritizing land-management interventions. The intervention should consider the dynamics of the rift, the area’s climatic conditions, and the human-induced drivers of soil erosion and land degradation. The findings of this study have vital socio-economic implications for maintaining agricultural productivity and enhancing food security by implementing sustainable land management and rehabilitation practices in tectonically active regions. Land degradation poses a significant threat to agricultural productivity and food security.

In contrast, sustainable land management practices help improve soil health, maximize crop productivity, and enhance the resilience of farming systems, generating long-term economic benefits in degradation-prone regions [1,13,98,99]. Importantly, the study clearly shows that the biological, chemical, and physical degradation of the area is not solely due to anthropogenic factors, but is also caused by high land surface temperatures and salinity, which reduce soil moisture and weaken the soil’s internal structure. Therefore, it suggests that soil erosion and land degradation in the rift area may result from geological and environmental interactions as well as human activities. Mechanistically, this broader vulnerability pattern arises because the composite LDV framework captures several interacting degradation pathways rather than water-driven soil loss alone. Salinity and sodicity can promote soil dispersion and surface sealing; vegetation and moisture stress can reduce protection and soil structural stability; elevated land surface temperature can intensify evaporation and drying; and hydrothermal alteration can weaken soil and weathered geological materials. Consequently, relatively flat areas with limited RUSLE-estimated water erosion may still exhibit substantial overall land degradation vulnerability when these chemical, biological, thermal, and geological stresses occur together.

These findings are broadly consistent with previous Ethiopian and international studies showing that land-use change, vegetation decline, rainfall erosivity, soil properties, salinity, sodicity, and thermal or moisture stress contribute to soil erosion and land degradation [11,34,81,82]. However, the present study differs in analytical scope by evaluating these processes within a geodynamically active, hydrothermally and salinity-stressed rift environment and by integrating biological, chemical, thermal, moisture-related, land-use, and RUSLE-derived physical erosion indicators within a single land degradation vulnerability framework. This broader integration demonstrates that areas with comparatively limited water-driven soil loss can still exhibit moderate-to-high overall degradation vulnerability where salinity, sodicity, hydrothermal alteration, vegetation stress, and elevated land surface temperature are pronounced. Thus, while the individual degradation mechanisms identified here are consistent with previous studies, their combined assessment provides a more rift-specific interpretation of land degradation than approaches focused primarily on land-use change or conventional RUSLE-based erosion assessment.

In addition, it provides insight into the development of hazardous gullies and landscape instability in rift regions. This enables stakeholders to identify areas with soil erosion and degradation risk, prioritize and implement cost-effective immediate interventions, and consider geological processes. The findings of this study also act as an early warning system to prevent the threat to agricultural lands posed by the expansion of soil erosion and irreversible degradation. It supports concerned bodies in developing land-use planning, programs, and policies for ecosystem conservation, continuous environmental rehabilitation, and climate-smart agriculture in the rift area. The study is also helpful in reducing future accumulation of eroded sediments in lakes, irrigation canals, and other reservoirs in the area. In the long run, it contributes more to the design of future land restoration and agricultural improvement strategies in rift areas to achieve sustainable development goals such as life on land (goal 15), no poverty (goal 1), and zero hunger (goal 2).

## 5 Limitation of the study

Despite its robust empirical framework and integration of multi-sensor datasets, this study presents several limitations. Primarily, the multi-criteria analytical design lacks an evaluation of temporal dynamics, such as multi-year climatic variability and continuous soil loss monitoring. Furthermore, while the model effectively captures geophysical and surface-physical driving factors, it does not incorporate local socio-economic drivers, such as land tenure frameworks, demographic pressures, agricultural market access, and community-level land management behaviors. Additionally, reliance on satellite-derived proxy indices (such as spectral salinity, NMDI, and LST) introduces inherent resolution constraints that may obscure microtopographic soil-crusting processes and fine-scale hydrothermal fluid venting. The support practice (P-factor) and cover management (C-factor) parameterizations within the RUSLE framework were largely derived from standardized regional land-use metrics, which may not fully capture dynamic community-led conservation measures or hyper-localized adaptive farming strategies. Future research should aim to integrate multi-decadal time-series monitoring with long-term socio-economic survey data to better constrain human-environment feedback loops and improve climate-adaptive land restoration policies across active rift ecosystems.

## 6 Conclusion and recommendations

This study assessed land degradation vulnerability in salinity- and hydrothermal-stressed regions of the Southern Main Ethiopian Rift using an integrated framework of multi-source Earth observation data and machine learning implemented on the Google Earth Engine platform. Conducted in a geodynamically active environment characterized by hydrothermal activity, salinity stress, and a human-modified landscape, the study highlights the importance of addressing soil erosion and land degradation to support sustainable agricultural productivity and effective land management.

Model validation demonstrated strong predictive performance, with an overall accuracy of 92.34%, a Kappa coefficient of 0.87, an F1-score of 0.95, and an ROC-AUC value of 0.9, indicating the reliability of the adopted approach for land degradation assessment and mapping. 92%. The LDV result revealed that about 48% of the study area is affected by moderate to very high levels of land degradation.

The findings emphasize the urgent need for both reactive and preventive interventions, including gully rehabilitation, sustainable land management practices, and informed land-use planning. The generated land degradation indicators and land degradation vulnerability maps provide crucial decision-support tools for policymakers, land managers, and agricultural practitioners by helping identify environmentally sensitive areas, guide agricultural expansion, support the selection of salt-and temperature-tolerant crops, and promote ecosystem restoration through reforestation and the establishment of resilient vegetation cover.

Long-term environmental sustainability in the Southern Main Ethiopian Rift requires coordinated efforts among researchers, government institutions, non-governmental organizations, and local communities. Public awareness programs, community-based conservation initiatives, and interdisciplinary research integrating agriculture, geology, and climate sciences are essential for enhancing ecological resilience and food security.

Despite its strengths, this study has some limitations. The analysis did not incorporate temporal assessments of soil erosion and land degradation dynamics due to limitations in multi-year climatic datasets and rainfall variability. In addition, socio-economic factors influencing land degradation were not considered. Therefore, future studies should integrate long-term environmental monitoring and socio-economic analyses to provide a more comprehensive understanding of land degradation processes and support more effective mitigation strategies.
